# DNA-PKcs promotes therapy resistance and metastatic recurrence in neuroblastoma

**DOI:** 10.1016/j.canlet.2026.218383

**Published:** 2026-02-27

**Authors:** Mahnaz Norouzi, Subin Kim, Beibei Zhu, Chi Wang, Natalie Wu, Katherine Somers, Eddy Shih-Hsin Yang, B. Mark Evers, Eric J. Rellinger, Piotr Rychahou

**Affiliations:** a Markey Cancer Center, University of Kentucky, Lexington, KY, USA; b Department of Surgery, University of Kentucky, Lexington, KY, USA; c Division of Cancer Biostatistics, University of Kentucky, Lexington, KY, USA; d Cincinnati Children’s Hospital Medical Center, Cincinnati, OH, USA; e Department of Radiation Medicine, University of Kentucky, Lexington, KY, USA

**Keywords:** DNA-PKcs, High risk neuroblastoma, Topoisomerase II inhibitors, Ionizing radiation, DNA double strand breaks, Apoptosis

## Abstract

**Purpose:**

High-risk neuroblastoma presents a serious clinical challenge with survival rates below 50%. Disease relapse most commonly occurs at distant metastatic sites and remains the primary driver of poor outcomes, emphasizing the need for therapies to target drivers of relapse.

**Experimental design:**

This study identified DNA-PKcs as a critical determinant of poor survival and metastatic relapse in neuroblastoma patients. We evaluated which therapeutic modality—chemotherapy or radiotherapy—when combined with DNA-PKcs inhibition, more effectively reduces metastatic burden and prevents recurrence.

**Results:**

Colony-forming assays revealed that established neuroblastoma colonies resist doxorubicin alone and require high-dose doxorubicin paired with DNA-PKcs inhibition to suppress progression. In contrast, low-dose radiotherapy in combination with DNA-PKcs inhibition effectively controlled colony progression. Maximal synergy between radiotherapy and DNA-PKcs inhibition was achieved when the inhibitor was administered within 4 h post-irradiation. Chronic co-exposure to doxorubicin and peposertib encouraged emergence of therapy-resistant cells, whereas chronic co-exposure to radiotherapy combined with peposertib disrupted neuroblastoma cells self-renewal and prevented long-term colony maintenance. In neuroblastoma metastases, adding DNA-PKcs inhibition to doxorubicin improved efficacy but induced gastrointestinal side effects and failed to eradicate tumors; pairing it with low-dose, fractionated radiotherapy resulted in total lesion regression, impaired tumor self-renewal, and prevented systemic adverse effects.

**Conclusions:**

Our findings correlate elevated DNA-PKcs levels with poor patient prognosis and show that low-dose radiotherapy combined with peposertib effectively abrogates neuroblastoma self-renewal compared to chemotherapy-based regimens, thereby implicating DNA-PKcs as a key mediator of metastatic relapse and supporting radiotherapy plus DNA-PKcs inhibition as a compelling therapeutic strategy for relapsed or refractory high-risk neuroblastoma.

## 1. Introduction

Neuroblastoma (NB) is the most common extracranial solid tumor of children, afflicting approximately 700 children per year and accounting for 15% of all childhood cancer deaths. NB is a heterogeneous disease characterized by significant variability in its clinical manifestations, histology, and clinical course ranging from spontaneous metastatic regression to overwhelming tumor burden despite intensive treatment regimens. Nearly half of children with NB are risk-stratified to having high-risk neuroblastoma (HR-NB) based upon age of diagnosis, extent of disease, histologic findings, and genetic features. Children with HR-NB have poor overall survival rates (~50%) despite aggressive multimodal therapy with metastatic disease progression and relapse being common causes of treatment failure [1,2]. Children with HR-NB are aggressively treated with induction chemotherapy, surgery, autologous stem cell transplantation, radiotherapy, and immunotherapy [3–5]. Unfortunately, treatment failure with the emergence of drug resistance in HR-NB patients is a major obstacle of modern-day treatment strategies [6,7]. Moreover, these patients are particularly susceptible to developing treatment-related complications as a result of high dosage or multiple cycles of chemotherapy [2,7–10]. These features highlight the need to optimize treatment response of current strategies and, if feasible, lessen drug toxicity to improve the quality of life for children with HR-NB.

Triggering DNA double-strand breaks (DSBs) is a common principle in modern treatment of HR-NB. Induction chemotherapy is the current upfront approach for treating children with intermediate- and high-risk NB. Regimens include agents like etoposide and doxorubicin, which induce cell death at least in part by generating DSBs. Radiation is also a critical component of consolidation therapy used for both post-operative locoregional control and treatment of residual metastatic sites. DSBs are the most lethal form of DNA damage, and the effective response of cancer cells to these cytotoxic effects depends on the activity of DNA damage response (DDR), a highly orchestrated signaling network that detects DNA lesions to trigger cellular responses. These responses involve DNA repair pathways and various other molecular pathways, including cell cycle arrest and apoptosis, which work together to repair DNA damage precisely, thereby preserving genome integrity [11–16]. In response to more severe DNA damage, DDR pathways initiate programmed cell death, or apoptosis, to kill damaged cells and avoid mutation accumulations. However, as cancer cells frequently carry DDR gene deficiencies, they have the ability to escape these mechanisms by triggering ectopic DNA repair systems, allowing them to repair damaged DNA and survive [15,17,18]. DNA repair activity is commonly associated with the development of drug resistance in tumor cells and targeting DNA repair pathways is an approach to increase the sensitivity of tumor cells to chemo-radiotherapeutic approaches [19–26]. It is now evident that targeted inhibition of DDR pathway components, like ATR, CHK1 and PARP inhibitors, in combination with chemo-radiotherapeutics is an effective strategy in targeted cancer therapies [27,28]. Putatively less toxic small molecule-based approaches that directly target DNA repair proteins have been discovered and are being evaluated in clinical trials for many cancers with the potential to revolutionize the future of cancer therapy [27–29]. Emerging data has revealed that genetic alterations in many DDR genes are associated with HR-NB, thus potentially serving as therapeutic approaches that may result in reducing the possibility of side-effects and the occurrence of drug resistance [29–31].

The DNA-dependent protein kinase catalytic subunit (DNA-PKcs) is a key component of the nonhomologous DNA end joining (NHEJ) DNA repair pathway and participates in the repair of DSBs induced by ionizing radiation and DNA damaging drugs, impairing tumor cell sensitivity to DSB-inducing therapies [32–34]. Clinical investigations have demonstrated that high expression of DNA-PKcs is associated with cancer progression and treatment failure in various cancer types including ovarian and prostate cancer [35–40]. DNA-PKcs is encoded by *PRKDC* gene. Within neuroblastoma, high *PRKDC* expression has been associated with poor overall survival using the Versteeg database featuring 88 human NBs [17]. This study demonstrated that DNA-PKcs blockade enhanced the sensitivity of NB cells to radiation *in vitro*. Intriguingly, a recent, independent CRISPR screen aimed at identifying synthetic lethal genetic vulnerabilities to common NB chemotherapies revealed DNA-PKcs as a critical mediator of doxorubicin-mediated resistance [41]. These two studies independently validate the role of DNA-PKcs in facilitating therapeutic resistance to DSB-inducing main-stays of high-risk NB therapy.

The focus of this study was to define role of DNA-PKcs in NB metastasis relapse and determine which therapeutic modality offers best outcomes in metastatic tumors regression and prevention of relapse. The cytotoxic consequences of both doxorubicin and radiation therapy in NB can be broadly attributed to the induction of DSBs providing a common thread of therapeutic efficacy and sensitization to DNA-PKcs blockade. Radiation generates predictable regionally defined DSBs while chemotherapy-based damage is stochastic and replication-associated. There is a critical need to translate and integrate these biologic vulnerabilities into modern-day treatment approaches and validate them in disease relevant models. In NB, metastatic niches—particularly bone marrow, bone, lymph nodes, and liver—are the predominant locations of relapse and resistance, characterized by enhanced DNA repair pathways [42]. We initially assessed *PRKDC* expression against high-risk clinical features and found it significantly upregulated in locally advanced and metastatic NB. The role of *PRKDC* in metastatic NB was further corroborated by our *in vivo* studies. DNA-PKcs inhibition improved the efficacy of doxorubicin therapy in suppressing BE(2)-C liver metastatic progression but was unable to fully eradicate metastatic tumors in the liver and its use was limited by gastrointestinal side effects. Conversely, DNA-PKcs inhibition in combination with radiotherapy effectively induced BE(2)-C liver metastases tumor regression, achieving complete clearance in liver regions with maximum radiation exposure—without causing gastrointestinal side effects. This research highlights the critical role of DNA-PKcs as a mediator of NB therapy resistance and relapse, and demonstrates combined radiotherapy and DNA-PKcs inhibition as a promising therapeutic approach for patients with high-risk, relapsed or refractory disease.

## 2. Results

### DNA-PKc is highly expressed within human NB tissues.

Prior assessment of DNA-PKcs expression have been focused on mRNA quantification using microarray analyses. We first sought to evaluate DNA-PKcs as a potential actionable target by examining protein expression in human NB using a commercially available tissue microarray (TMA; NB642c). This array comprises 27 NB patients representing multiple anatomic sites associated with the sympathetic nervous system, including the retroperitoneum (16 cases), adrenal gland (5 cases), mediastinum (4 cases), and pelvic cavity (2 cases). Two tumors demonstrated invasion into adjacent organs, including the liver or spleen. Histopathologic subtypes included undifferentiated (n = 10), poorly differentiated (n = 13), differentiated (n = 3), and ganglio-neuroblastoma, nodular type (n = 1). INSS staging indicated that most tumors (23 of 27, 85%) were early stage (Stage I), with one case each of Stage II and Stage IIB, and two cases of Stage IV. The array also includes 10 cores from five peripheral nerve samples, which served as normal controls. Data on treatment history, primary versus metastatic origin, TNM classification, and clinical outcomes were not available.

**Fig. 1.**
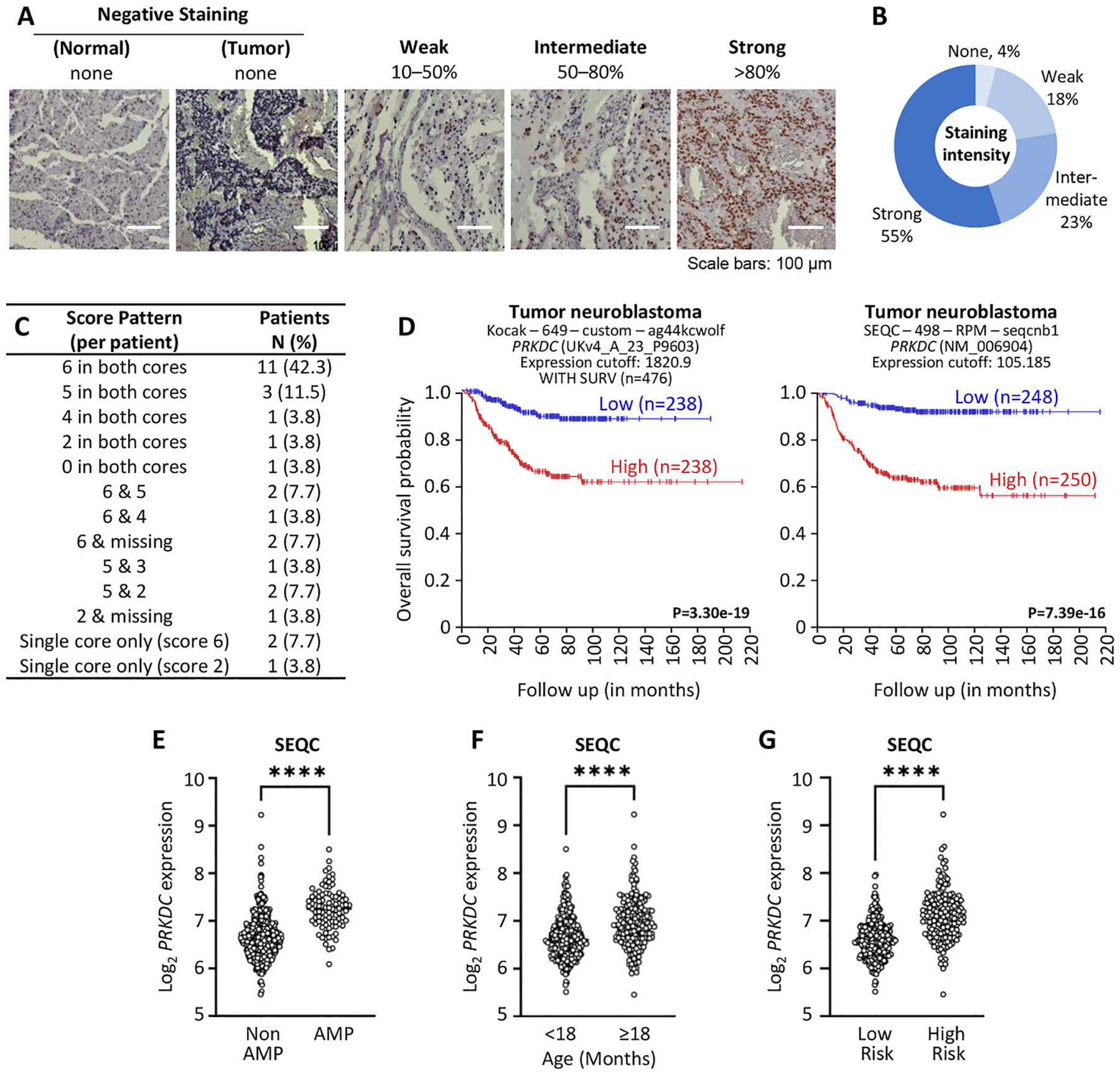
*PRKDC* gene expression is associated with high-risk NB and survival outcomes. **(A)** Microscopic features of the immunohistochemical staining of DNA-PKcs in NB TMAs (NB642c). (B) Intensity of DNA-PKcs IHC staining in TMA cores (n = 49), categorized as none (0%), weak (<50%), intermediate (50–80%), or strong (>80%). (C) The semi-quantitative scoring data demonstrate that the majority of patients exhibited high DNA-PKcs expression, achieving the maximum score in both cores. (D) Kaplan–Meier analysis shows that high *PRKDC* gene expression levels correlate with poor overall survival in NB patients (Kocak, n=649, SEQC n=498). (E) *PRKDC* expression in NB tumors with *MYCN* amplification. (F) *PRKDC* expression in NB patients by age group. (G) *PRKDC* gene expression in HR-NB compared to low-risk cases; ****p<0.0001.

Immunostaining data were unavailable for five cores, primarily due to tissue loss during staining. In total, samples from 26 patients were analyzed (22 patients with two cores and 3 patients with a single core). Immunohistochemical (IHC) analysis, performed by a pathologist, revealed strong DNA-PKcs protein expression in 55% of cores (27 out of 49, corelating with 16 patients), intermediate staining in 23%, weak staining in 18%, and absent expression in 4% of evaluable cores (Fig. 1A and B). A semi-quantitative scoring system was applied by summing the proportion of positive cells with staining intensity, yielding a maximum score of 6. Using this system, 11 patients achieved the highest score (6) in both cores (42.3%). This was followed by three patients who scored 5 in both cores (11.5%), one patient who scored 4 in both cores (3.8%), one patient who scored 2 in both cores (3.8%), and one patient in whom DNA-PKcs expression was not detected (score = 0, 3.8%). Among the cases with only one core or with discordant scores between cores, five patients scored 6 in one core; of these, two patients scored 5 (7.7%), and one patient scored 4 (3.8%) in the second core. Two patients had the second core missing (7.7%). In addition, three patients scored 5 in one core; in these cases, the second core scored 3 in one patient (3.8%) and 2 in two patients (7.7%). Finally, one patient was represented by a single core, which scored 2, while the other core was missing (3.8%, Fig. 1C). These data validate that DNA-PKcs are highly expressed within most human NBs.

### High *PRKDC* is associated with poor survival and adverse clinical risk factors in NBs including Advanced Stage of Disease.

We next sought to expand the prior analyses of Dolman et al. to larger publicly available NB datasets including the Kocak [43] and SEQC (GEO ID: GSE49710) to define the associations with overall survival and adverse clinical risk factors including age at diagnosis, *MYCN*-amplification, risk-stratification, and stage of disease. Kaplan-meier analysis was performed using median cutoff for both the SEQC and Kocak datasets using R2 Genomics Analysis and Visualization Platform (https://hgserver1.amc.nl/cgi-bin/r2/main.cgi). Survival curves within both datasets revealed that high *PRKDC* is associated with poor overall survival in NB patients (p < 0.001; Fig. 1D).

**Fig. 2.**
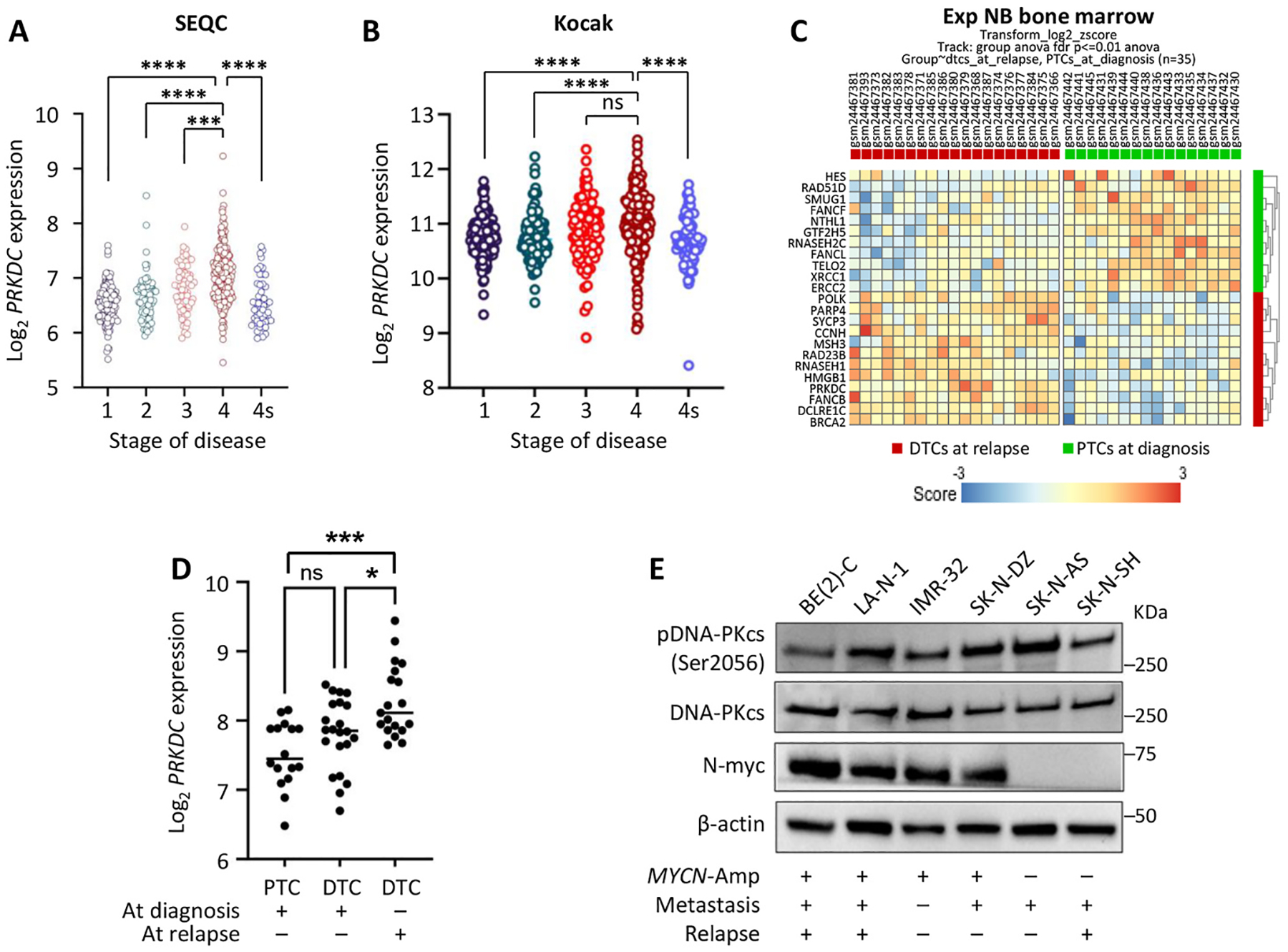
*PRKDC* genes expression correlated with adverse clinical risk factors in NBs. (A, B) Correlation between PRKDC gene expression levels and NB tumor progression to stage 4. (C) Heatmap of differential expression of DNA replication and repair pathway (KEGG) genes in PTCs (green) at diagnosis and DTCs (red) at relapse. Color intensity represents expression level. Heatmap of KEGG pathways involved in DNA replication and repair in primary tumor cells (PTCs) at diagnosis (green) and in disseminated tumors cells (DTCs) at relapse (red). (D) PRKDC gene expression in PTCs at diagnosis, DTCs at diagnosis and in DTCs at relapse (Ambros study, n=86) revealed a significant upregulation of PRKDC in DTCs at relapse. (E) Western blot analysis in MYCN-amplified (BE(2)-C, LAN-1, IMR-32 and SK-N-DZ) and MYCN-non-amplified (SK-N-AS, SK-N-SH) NB cell lines (*p<0.05, ***p<0.001, ****p<0.0001).

Older age (>18mos at diagnosis) and *MYCN*-amplification are key contributors to modern-day neuroblastoma risk stratification. Univariate analysis of *PRKDC* xpressions within the SEQC database revealed that *PRKDC* is enriched within both older children (>18mos of age) and children with tumors featuring *MYCN*-amplification (p < 0.0001; Fig. 1E and F). These features contribute to the COG (Children’s Oncology Group) risk stratification, and notably, increased *PRKDC* expression was associated with high-risk tumors (p < 0.001; Fig. 1G). Correlation between DNA-PKcs expression levels and NB tumor stage was also analyzed. *PRKDC* expression showed a significant increase in higher NB stages within the SEQC and Kocak databases. Within the SEQC database, increased *PRKDC* expression was observed within Stage 4 tumors compared to all other stages (St1,2,4S vs. 4; p < 0.0001 and St 3 vs. 4; p = 0.003; Fig. 2A). A similar trend was observed within the Kocak database with increased *PRKDC* expression observed between St1,2,4S vs. St 4 (p < 0.0001). However, no significant difference was observed with comparison between St 3 and St4 disease within this dataset (Fig. 2B). Overall, these findings demonstrate the *PRKDC* expression is associated with poor clinical risk factors including older age at diagnosis (>18mos), *MYCN*-amplification, advanced stage of disease and COG high-risk stratification.

### DNA Damage Response including DNA-PKcs Expression is Enhanced within Relapsed NB metastases.

The association of *PRDKC* expression with advanced stage NBs prompted us to evaluate whether *PRKDC* is enriched within the metastatic niche of human NBs. Rifatbegovic et al. found that GD2-enriched bone-marrow DTCs exhibit increased DNA-repair activity and reduced differentiation, changes that intensify at relapse and implicate therapy resistance and metastatic progression [42]. We leveraged this dataset to determine whether the DNA damage response and specifically DNA-PKcs was enriched between primary tumors, DTCs at diagnosis, and DTCs isolated at relapse.

Differential expression analysis using the R2 Genomics platform identified 23 genes out of 234 curated from KEGG DNA replication and repair pathways—including base excision repair (BER), Nucleotide excision repair (NER), Fanconi anemia (FA), mismatch repair (MMR), HR, and NHEJ—that were significantly altered between DTCs at relapse and primary tumor cells at diagnosis (PTC, FDR ≤0.01, Fig. 2C). Of these 23 differentially expressed genes, 18 exhibited a fold change greater than 1.5, encompassing both up- and downregulated genes, highlighting a substantial subset of transcriptional changes across multiple DNA repair pathways. Functional annotation revealed that these genes form interconnected DNA repair networks. Of interest, high-fidelity repair genes, such as those involved in HR and FA (*FANCF*, *RAD51D*, *FANCL*, *NTHL1*), were generally more highly expressed in tumor cells at diagnosis. In contrast, error-prone repair genes, including those associated with NHEJ and translesion synthesis (TLS), such as *PRKDC*, *POLK*, *DCLRE1C*, and *PARP4*, were specifically upregulated in DTCs at relapse. These changes are functionally interconnected and reflect a shift in DNA repair programs during relapses, potentially contributing to genomic plasticity and therapy resistance. As shown in Fig. 2D, expression data analysis from this study revealed a significant upregulation of *PRKDC* gene expression in DTC cells at relapse (n = 19) compared to their corresponding DTC cells at diagnosis (n = 22). Moreover, gene expression analysis revealed a more pronounced upregulation of *PRKDC* in DTC cells at relapse compared to primary tumor cells (PTCs, n = 16) at diagnosis (p < 0.001). Together, these findings demonstrate that DNA-PKcs is enhanced within the metastatic niche of NB DTCs with the highest levels of expression noted within the DTCs analyzed at relapse, suggesting that *PRKDC* expression is further enriched within relapsed DTCs that are a common driver of poor long-term survival. Moreover, Western blot analysis and confocal imaging confirmed expression of DNA-PKcs and pDNA-PKcs (Ser2056) in four *MYCN*-amplified (BE(2)-C, LAN-1, IMR-32, and SK-N-DZ) and two *MYCN*-non-amplified (SK-N-AS, SK-N-SH) NB cell lines (Fig. 2E; Supplemental Figure 1A).

### DNA-PKcs activity reduces the cytotoxic effect of Top II inhibitors on human NB cells.

First-line treatment for intermediate- and high-risk NB utilizes DNA-damaging agents like topoisomerase (Top) II inhibitors etoposide and doxorubicin. Given the critical role of DNA-PKcs in the DNA damage response and overall cell survival [22,37, 38], we investigated whether DNA-PKcs activity diminishes the sensitivity of NB cancer cells to the DNA-damaging chemotherapy agents etoposide and doxorubicin. Focusing on MYCN-amplified NBs, we selected BE(2)-C as a well-established model of aggressive disease and included SK-ND-Z as an additional MYCN-amplified line that harbors an 11q deletion associated with homologous recombination deficiency, allowing evaluation across distinct genetic backgrounds. Our assessment showed that proliferation of both SK-ND-Z and BE(2)-C cell lines decreased in response to doxorubicin and etoposide treatment in a dose-dependent manner; however, SK-ND-Z cells exhibited greater sensitivity to both agents. (Supplemental Fig. 1B and C). The calculated IC_50_ for BE(2)-C cells for etoposide and doxorubicin was 2.78 ± 0.16 μM and 0.44 ± 0.01 μM, respectively. The IC_50_ values dropped significantly in combination with 1 μM peposertib to 0.76 ± 0.1 and 0.20 ± 0.006 μM, respectively. Etoposide and doxorubicin showed potent inhibitory effects on SK-N-DZ cell viability, with IC_50_ values of 0.49 ± 0.02 μM and 51.87 ± 1.95 nM, respectively. A significant reduction, in the IC_50_ values (0.11 ± 0.01 μM and 30.27 ± 3.9 nM) was observed when SK-N-DZ cells were treated with each drug in combination with peposertib (Supplemental Fig. 1C and B).

**Fig. 3.**
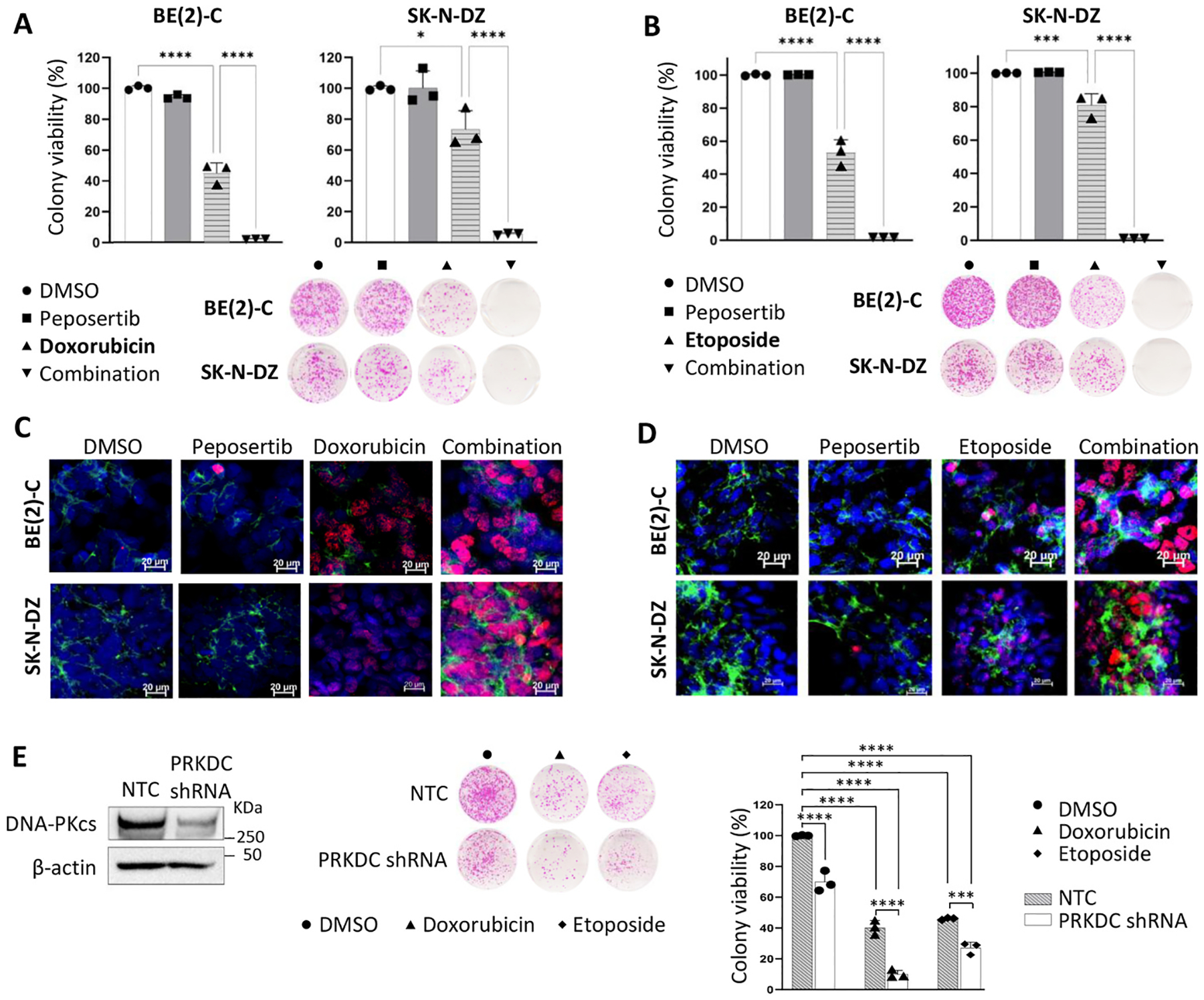
Inhibition of DNA-PKcs potentiates the cytotoxic effects of topoisomerase II inhibitors on NB cells in vitro. (A, B) BE(2)-C and SK-N-DZ cells were treated with doxorubicin (A) or etoposide (B), either individually or in combination with peposertib. Clonogenic assay was performed to evaluate long-term cell viability of NB cells. BE(2)-C and SK-N-DZ cells were treated with peposertib (1000 nM), and doxorubicin (BE(2)-C, 75 nM; SK-N-DZ, 10 nM) and etoposide (BE(2)-C, 500 nM; SK-N-DZ, 100 nM). Drugs were removed and replaced with fresh media 48 h later, and colonies were allowed to grow for 14 days. (C, D) DNA damage following doxorubicin (C) or etoposide (D) therapy in combination with peposertib was evaluated in BE(2)C and SK-N-DZ using immunofluorescence staining of γ-H2AX (Ser139) (Red: γ-H2AX; Blue: DAPI; Green: Phalloidin). (E) Clonogenic assay was performed to evaluate long-term viability of NB cells. BE(2)-C NTC and PRKDC knockdown cells were treated with etoposide (500 nM) and doxorubicin (75 nM). Drugs were removed and replaced with fresh media 48 h later, and colonies were allowed to grow for 14 days; *p<0.05, ***p<0.001, ****p<0.0001.

To assess cell reproductive death after treatment with etoposide or doxorubicin, we conducted a clonogenic assay. Both BE(2)-C and SK-N-DZ cells exhibited significantly reduced survival upon treatment with etoposide and doxorubicin. Echoing the findings from our proliferation studies, the addition of peposertib further diminished cell viability. Moreover, the markedly decreased colony formation when either chemotherapeutic agent was combined with peposertib underscores the notion that DNA-PKcs plays a critical role in mediating chemoresistance in NB cells (Fig. 3A and B).

DNA repair inhibitors compromise the cell’s intrinsic ability to repair DNA damage, causing an accumulation of DSBs and thereby intensifying the genotoxic effects of DNA-damaging agents. To determine whether the effect on NB viability from combination therapy stems from exacerbated DNA damage, we evaluated the phosphorylation of γ-H2AX (Ser139), a marker for DSBs. As illustrated in Fig. 3C, the addition of peposertib to doxorubicin resulted in a substantial increase in γ-H2AX foci formation. Similarly, a 24-h treatment with 2 μM etoposide in BE (2)-C cells and 0.5 μM in SK-N-DZ elicited DSB induction in both cell lines (Fig. 3D). The role of DNA-PKcs expression in NB cell survival was also confirmed with *PRKDC* gene knockdown. *PRKDC* knockdown (Fig. 3E) alone or in combination with chemotherapy resulted in significant decrease in viability compared to the NTC group (p < 0.0005). The survival rate of *PRKDC* knockdown with chemotherapy combination decreased below 30% in response to treatment with both etoposide (0.5 μM) and doxorubicin (75 nM) (p < 0.001).

To elucidate the mechanism underlying the chemo-sensitizing effects in combination groups, we evaluated key markers of apoptosis and cell cycle regulation. As shown in Supplemental Fig. 2A and B, treatment with doxorubicin or etoposide alone significantly elevated cleaved PARP protein levels while reducing cyclin D1 expression in both BE(2)-C and SK-N-DZ cell lines after 48 h. Doxorubicin therapy alone resulted in a strong induction of cleaved PARP expression and a decrease in cyclin D1 protein levels 48 h after treatment with doxorubicin 500 and 250 nM in BE(2)-C and SK-N-DZ cells, respectively. Considering the significant increase in DSBs, we further analyzed whether the increased DNA damage induced by combination treatment is the molecular consequence of activated programmed cell death pathways. We measured the cytoplasmic histone-associated DNA fragments using a Cell Death ELISA assay after 48 h of treatment with etoposide or doxorubicin alone and in combination with peposertib. DNA fragmentation analysis in BE(2)-C and SK-N-DZ cells confirmed DNA fragmentation and subsequent apoptosis in doxorubicin and in combination with peposertib in both cell lines (p < 0.0001) (Supplemental Fig. 2C and D).

### DNA-PKcs inhibition enhanced apoptosis and suppressed liver metastasis progression in combination treatment with doxorubicin.

Stage 4 NB in children over 18 months of age remains a significant challenge, as approximately two-thirds of these patients either fail to respond to initial treatments or experience disease progression or relapse after an initial response [44]. In this study, we evaluated the response of metastatic tumors to doxorubicin chemotherapy in conjunction with DNA-PKcs inhibition, recognizing that the metastatic response to induction therapy is a critical prognostic indicator in HR-NB [45–47]. To investigate the therapeutic efficacy of doxorubicin against BE(2)-C liver metastases, we tested four distinct treatment regimens [46–48]. In our initial approach, doxorubicin was administered at doses of 0.25 or 0.75 mg/kg twice weekly; however, this regimen failed to inhibit the progression of liver metastases (Supplemental Fig. 3A). We then increased the dosing frequency to three times per week at 0.75 mg/kg in combination with peposertib but did not observe significant therapeutic effect on metastatic burden. Given that doxorubicin’s effectiveness in metastatic tumors is largely dependent on its ability to induce DNA damage, we concluded that doses below 1 mg/kg were insufficient to substantially damage DNA and arrest metastatic progression (Supplemental Fig. 3B). Therefore, as a next step, we escalated the doxorubicin dose to 2.5 mg/kg, administering it for three consecutive days followed by a 3-day break. The higher dose regimen did not improve the combination groups’ ability to suppress metastatic progression (Supplemental Fig. 3C) and instead led to significant gastrointestinal toxicity, evidenced by abdominal distension and weight loss.

**Fig. 4.**
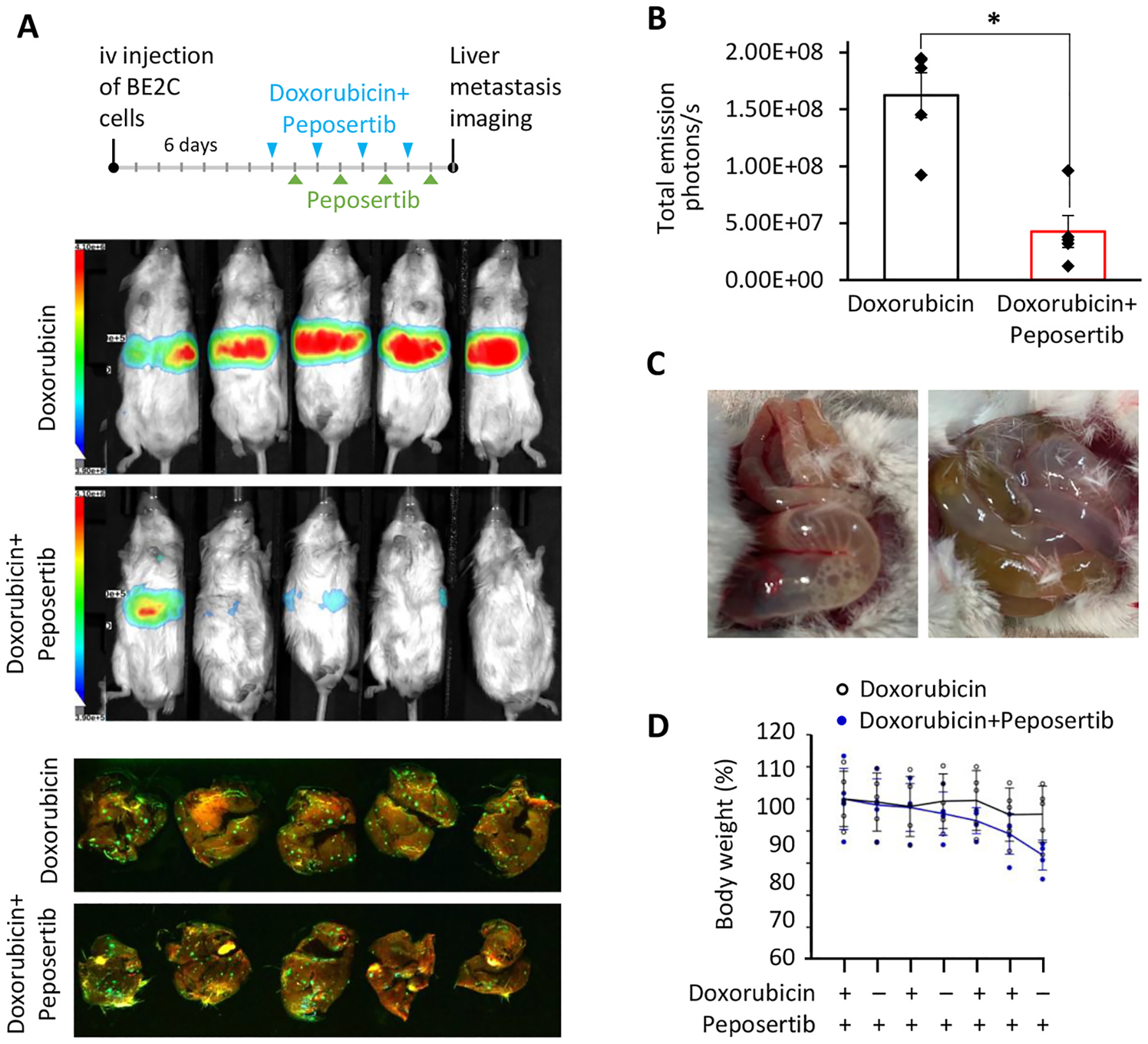
DNA-PKcs inhibition in combination with doxorubicin suppresses liver metastasis progression. (A) BE(2)-C GFP-Luc cells were intravenously injected into NOD rag gamma mice. Chemotherapy commenced 6 days post-injection, with q.o.d doxorubicin (2.5 mg/kg, iv) and peposertib (100 mg/kg, gavage) as combination therapy, followed by a single dose of peposertib alone (100 mg/kg) the next day. Bioluminescent imaging (top) and GFP imaging (bottom) to demonstrate therapy outcomes on liver metastasis burden. (B) Bioluminescent signal quantification was used to measure liver metastasis burden at the end of the study (*p<0.05). (C) Gross photograph of GI track in doxorubicin and peposertib combination. (D) Mouse weight.

We hypothesized that inhibition of DNA-PKcs by peposertib may not be optimally timed relative to the peak DNA damage induced by doxorubicin. By the time cancer cells encounter significant genotoxic stress, the inhibitory effect of peposertib might have already diminished. Thus, our next step was to determine whether a modified regimen—featuring reduced doxorubicin frequency coupled with increased DNA-PKcs inhibition—could effectively induce cell death in established NB liver metastases. Fourteen days after the injection of cancer cells, BE(2)-C liver metastases were treated with doxorubicin administered every other day alongside peposertib (n = 2), followed by peposertib monotherapy (n = 2). Immunohistochemical analysis of the metastatic tumors revealed increased apoptotic response in the combination treatment group, evidenced by cleaved caspase staining (Supplemental Fig. 3D). These findings suggested that increasing the frequency of peposertib administration might more effectively synergize with the DNA damage induced by doxorubicin. Accordingly, in our subsequent study, doxorubicin was administered every other day alongside peposertib, followed by a phase of peposertib monotherapy (Fig. 4A). In an effort to minimize direct gastrointestinal exposure, doxorubicin was administered intravenously. This regimen resulted in increased response to doxorubicin therapy in the combination group (Fig. 4B), but still failed to alleviate gastrointestinal toxicity, evidenced by abdominal distension and weight loss (Fig. 4C and D). Immunohistochemical analysis confirmed that the combination therapy did not induce toxic effects in the liver, lungs, or heart that could account for the deterioration in the animals’ condition (Supplemental Fig. 4A). These findings reveal that DNA-PKcs inhibition markedly intensifies the gastrointestinal toxicity of systemic DNA-damaging therapies like doxorubicin, thereby narrowing the therapeutic window. A promising alternative strategy to reduce this toxicity without sacrificing anticancer efficacy is the targeted delivery of chemotherapeutic agents with a nanocarrier or the use of precision-focused modalities—such as XRT or proton therapy.

### DNA-PKcs inhibition in combination with radiotherapy eliminates BE(2)-C liver metastasis in high-dose regions.

**Fig. 5.**
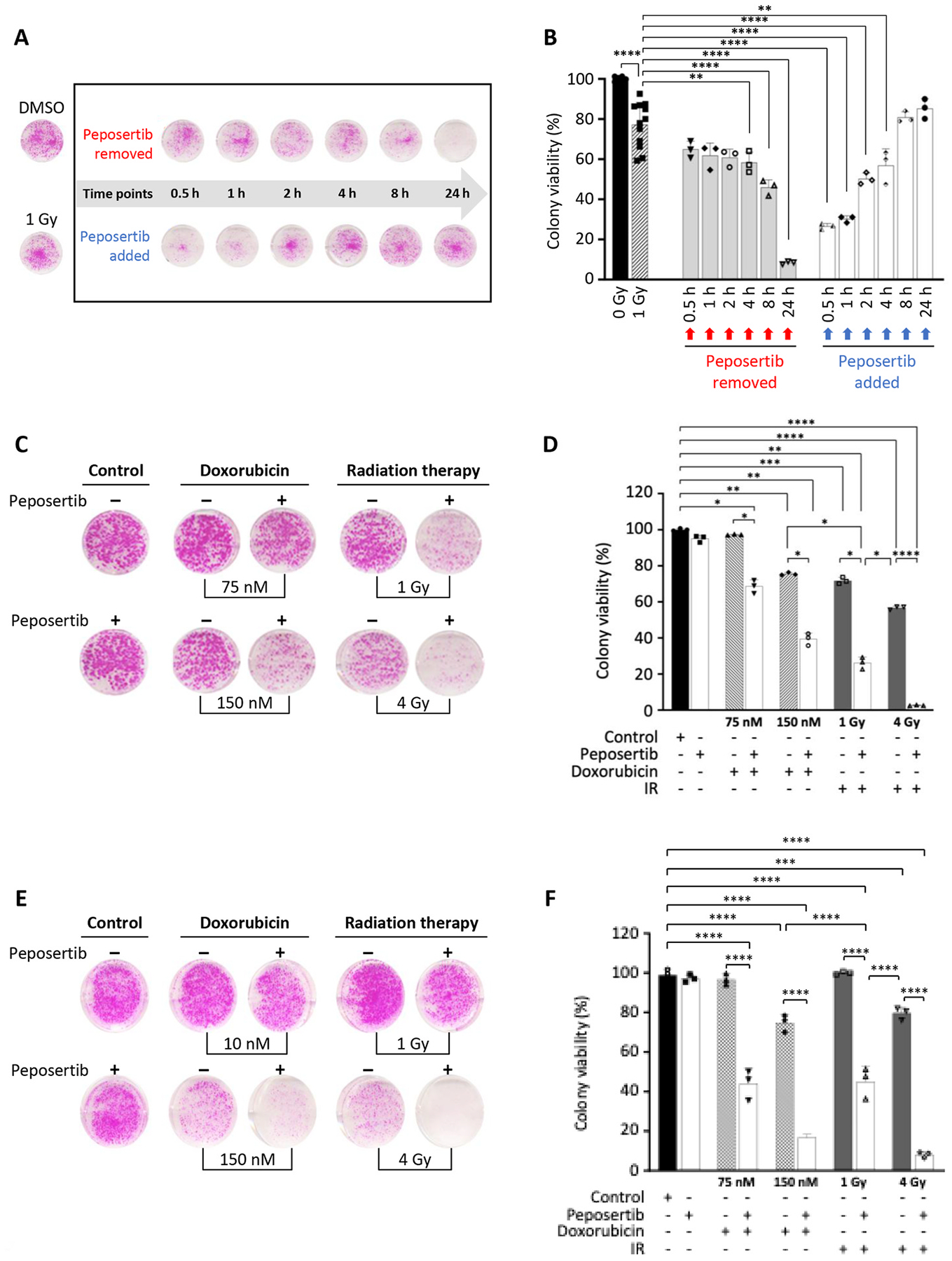
Optimizing the timing of DNA-PKcs inhibition in NB radiation therapy is crucial for enhancing treatment efficacy. (A) BE(2)-C cells were pre-treated with peposertib for 30 min before IR, then cells were irradiated at 1 Gy and peposertib was removed at different time points (top panel). BE(2)-C cells were irradiated at 1 Gy and peposertib was added at different time points post-IR (bottom panel). (B) Colony viability was measured by colony formation assay. (C) BE2C cells were cultured for 7 days to form colonies before initiating doxorubicin (75 and 150 nM) or IR (1 Gy and 4 Gy) therapy alone or in combination with peposertib. Following treatment, the media was refreshed 48 h later, and the colonies were permitted to grow for an additional 7 days. (D) Colony viability was measured by colony formation assay. (E) Similarly, SK-N-DZ cells were allowed to form colonies for 7 days prior to treatment with doxorubicin (10 or 20 nM) or ionizing radiation (0.5 Gy or 2 Gy), alone or in combination with peposertib. The culture medium was refreshed 48 h post-treatment, and colonies were incubated for an additional 7 days. (F) Represents the Colony viability measurement.; *p<0.05, **p<0.01, ***p<0.001, ****p<0.0001.

Radiotherapy is a vital component of the therapeutic regimen for HR-NB that is used to improve post-operative local control, treatment of residual metastatic sites, or for palliation [1,6]. Next, we evaluated whether integrating radiotherapy-induced localized DNA damage with DNA-PKcs inhibition could mitigate the adverse side effects we observed with systemic DNA-damaging therapies. First, we hypothesized that the timing of DNA-PKcs inhibition is a critical determinant of the success for combination radiotherapy. To test this hypothesis and optimize the use of DNA-PKcs inhibitors, we evaluated two therapeutic approaches. In the first strategy, BE(2)-C cells were treated with peposertib prior to radiation, and the drug was removed at multiple time points post-irradiation (0.5, 1, 2, 4, 8, and 24 h). In the second strategy, BE(2)-C cells were irradiated first and subsequently treated with peposertib at different time points. Colony formation was compared with controls, either treated with DMSO without irradiation or irradiated in the absence of peposertib (IR-only). The time of irradiation was defined as time zero. After the longest peposertib treatment interval (24 h), culture media were replaced for all treatment groups, including control and IR-only conditions, and cells were subsequently cultured under identical conditions for an additional 13 days before being fixed and stained simultaneously at a common endpoint. Our findings indicate that insufficient drug exposure after irradiation diminished the effectiveness of combination therapy, with the strongest therapeutic impact achieved within a 24-h post-irradiation window (Fig. 5A and B). This experiment underscored the necessity of sustaining cancer cell exposure to peposertib for at least 4 h post-irradiation to maximize therapeutic benefit (Fig. 5A and B). These findings emphasize the necessity of achieving DNA-PKcs inhibition within 4 h of radiotherapy to optimize combination therapy outcomes. Moreover, our data suggest that extending peposertib administration beyond a 24-h window is likely unnecessary, thereby reducing patient exposure without compromising therapeutic efficacy.

The success of metastatic tumor therapy depends on treatments that effectively disrupt the ability of cancer cells to proliferate and sustain themselves, ultimately leading to tumor regression [49]. Therefore, for combination radiotherapy to be effective, it must not only target individual cancer cells but also eradicate established micrometastases. To model this, we employed a clonogenic assay, allowing cells to form stable colonies, representing micrometastatic growth. BE(2)-C cell colonies were established for 7 days prior to administering either doxorubicin (75 nM or 150 nM) or radiation (1 Gy or 4 Gy) in combination with peposertib. As shown in Fig. 5C, the previously effective dose of doxorubicin (75 nM) did not significantly impact the viability of established BE(2)-C colonies. However, combining doxorubicin (75 nM) with peposertib or doubling the doxorubicin dose alone resulted in a modest decrease in colony survival (32% and 25%, respectively, Fig. 5D). Notably, the combination of doxorubicin (150 nM) and peposertib resulted in a 61% reduction in established NB colonies, aligning with our *in vivo* findings that confirm the therapeutic efficacy of this combination. However, its full potential is constrained by the challenge of delivering a sufficiently high dose without inducing significant side effects. Combination radiotherapy at a low 1 Gy dose proved more effective than high-dose doxorubicin in promoting regression of established colonies, reducing colony viability by 74% (Fig. 5D). Increasing the irradiation dose to 4 Gy further amplified this effect, leading to a 43% reduction in the ionizing radiation (IR) monotherapy group and a striking 97.5% decrease in the combination treatment group (Fig. 5D). To further validate the effectiveness of the peposertib combination, we tested the same model in SK-N-DZ cells (Fig. 5E). SK-N-DZ colonies that had been allowed to grow for 7 days prior to treatment completely lost sensitivity to low-dose doxorubicin (10 nM), with approximately 96% of cells remaining viable one week after treatment Fig. 5E). A similar effect was observed with low-dose irradiation, although 0.5 Gy IR effectively abolished colony formation in a standard clonogenic assay (data not shown), established colonies maintained nearly 100% viability (Fig. 5E). Notably, combining peposertib with either low-dose doxorubicin (10 nM) or low-dose IR (0.5 Gy) significantly reduced colony viability to approximately 45% in both treatment groups. Increasing doxorubicin to 20 nM modestly lowered colony survival to 75%; however, when combined with peposertib, the same concentration, reduced established SK-N-DZ colonies by nearly 80%. Similarly, increasing the IR dose resulted in only a 20% reduction in the viability of established SK-N-DZ colonies, whereas combining 2 Gy IR with peposertib markedly decreased viability by approximately 90% (Fig. 5 F). Together, these findings confirm that neuroblastoma cells rely on DNA-PKcs activity for survival under genotoxic stress.

Effective clearance of metastatic tumors in affected organs necessitates apoptosis induction and combining radiotherapy with DNA-PKcs inhibition enhanced c-PARP expression while downregulating cyclin D1 expression (Supplemental Fig. 4B). These *in vitro* observations further support selection of radiotherapy as a more favorable approach with a higher therapeutic index when combined with DNA-PKcs inhibition.

**Fig. 6.**
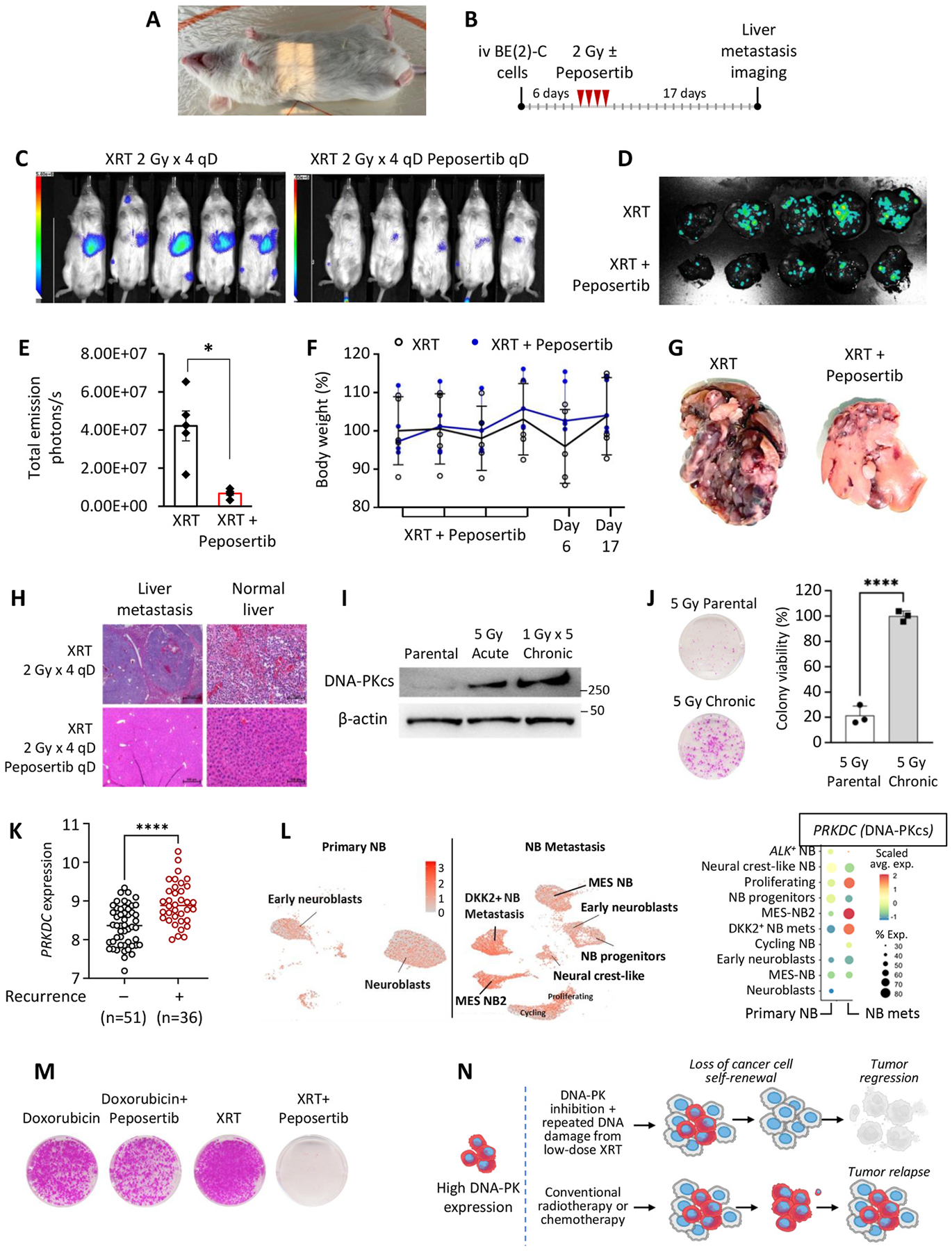
DNA-PKcs inhibition in combination with radiotherapy results in elimination of BE(2)-C liver metastasis in areas with the highest IR exposure. **(A)** Area of irradiation outlined by a light window. **(B)** BE(2)-C GFP-Luc cells were injected iv into NOD rag gamma mice. Radiation therapy started 6 days after cancer cell injection with daily 2 Gy irradiation alone or in combination with peposertib (gavage; 100 mg/kg) for 4 days. **(C)** Bioluminescent imaging and **(D)** GFP imaging demonstrate therapy outcomes on liver metastasis burden. **(E)** Bioluminescent signal quantification to measure liver metastasis burden at the end of the study (*p < 0.05). **(F)** Mouse weight. **(G)** Gross photograph of liver metastasis. **(H)** H&E imaging of liver metastasis and normal liver adjacent to metastatic tumors. **(I**) DNA-PKcs expression in surviving colonies after acute (5 Gy) and chronic (1 Gy × 5 times) IR exposure. **(J**) BE(2)-C cells subjected to repeated low-dose irradiation (1 Gy × 5) exhibited increased resistance to acute high-dose radiotherapy (5 Gy) relative to the parental cell line. **(K**) *PRKDC* gene expression in NB tumors with recurrence or progression (Versteeg study, n = 87) revealed a significant upregulation of *PRKDC* in tumors at relapse. **(L)** Side-by-side UMAP visualization of NB tumor cell subclusters in post-treatment neuroblastoma at primary (n = 21,248 cells) and metastatic (n = 24,526 cells) sites after re-clustering. Colors represent assigned cell types within NB tumors. The dot plot shows *PRKDC* gene expression in tumor subpopulations in NB at primary and metastatic locations post-induction treatment. The color scale represents scaled average expression of *PRKDC* in each tumor type, and the size of the circle indicates the proportion of cells expressing *PRKDC*. **(M)** DNA-PKcs inhibition in combination with radiotherapy disrupts self-renewal capacity of BE(2)-C cells. BE(2)-C cells were treated with 5 cycles of doxorubicin (50 nM) therapy or irradiation (1 Gy) alone or in combination with peposertib. **(N)** Elevated DNA-PKcs expression in metastatic NB cells confers a survival advantage under chemotherapeutic and radiotherapeutic stress. DNA-PKcs inhibition combined with repeated low-dose ionizing radiation impairs cancer cell self-renewal capacity and suppresses tumor relapse.

In our next experiment, we evaluated the efficacy of radiotherapy in treating NB metastases *in vivo* [25,26]. Radiation therapy for BE(2)-C cell liver metastases began 6 days post-injection, allowing sufficient time for the establishment of liver micrometastases. During treatment, mice were positioned on their backs, and the irradiation area was precisely defined using a light-guided window (Fig. 6A). Peposertib was administered via gavage 30 min before each radiotherapy session, which was delivered at 2 Gy per day for four consecutive days (Fig. 6B). The combination of radiotherapy and peposertib significantly suppressed NB liver metastasis progression (Fig. 6C–E) while remaining well tolerated, with no observed complications, gastrointestinal side effects, or toxicity (Fig. 6F). Most strikingly, areas receiving the highest doses of radiotherapy were entirely devoid of metastatic tumors, as confirmed by both gross examination and microscopic analysis (Fig. 6G). Combination radiotherapy effectively suppressed the aggressive behavior of surviving metastatic tumors, limiting their expansion into adjacent healthy liver tissue, as evidenced by the preservation of normal liver structures near metastatic tumors (Fig. 6H). In contrast, the radiotherapy-alone group exhibited extensive metastatic tumor burden and infiltration into normal liver, leading to significant hepatic cell damage and erythrocyte infiltration. The pronounced regression of metastatic tumors in regions receiving maximal irradiation represents a striking observation, suggesting that combination radiotherapy may constitute an effective strategy for preventing the progression and recurrence of metastatic NBs.

To investigate the role of DNA-PKcs in NB relapse following radiation therapy, parental BE(2)-C cells were subjected to either acute (5 Gy) or repeated low-dose (1 Gy × 5) irradiation. Surviving colonies were analyzed by western blotting, revealing a marked upregulation of DNA-PKcs expression in both treatment groups, with the highest expression detected in cells exposed to chronic irradiation (Fig. 6I). As illustrated in Fig. 6J, colonies exposed to chronic irradiation developed resistance to subsequent high-dose radiotherapy. Recurrent and refractory tumor lesions remain a major challenge in the treatment of patients with HR-NB [50]. As shown in Fig. 6K, a significantly higher expression of the *PRKDC* was also detected in patients with recurrent and progressive disease (n = 36) based upon gene expression data analysis from the Versteeg study [51]. We performed a comparative single-cell RNA-seq analysis of primary versus metastatic NB lesions that revealed selective up-regulation of *PRKDC* in distinct metastatic cell clusters (Fig. 6L). Notably, the highest enrichment occurred in mesenchymal-like MES-NB2 cells, proliferating NB subsets, and DKK2+ NB populations— suggesting DNA-PKcs’s role in sustaining those metastasis-driving subpopulations of NBs.

Given the elevated DNA-PKcs expression observed in irradiation–resistant colonies, patients with recurrent and metastatic disease, we investigated whether DNA-PKcs inhibition could disrupt the self-renewal capacity of NB cells following repeated low-dose chemotherapy or radiotherapy. BE(2)-C were subjected to either a single dose of doxorubicin (50 nM) or ionizing radiation (1 Gy), administered alone or in combination with peposertib. Surviving colonies were subsequently subjected to four additional cycles of the same treatment regimen. Notably, colonies derived from the 1 Gy/peposertib combination group exhibited a near-complete loss of self-renewal capacity following repeated exposure, effectively preventing relapse within our *in vitro* model (Fig. 6M). In contrast, clonogenic assays demonstrated that colonies treated with doxorubicin, with or without peposertib, retained their ability to reform colonies after five treatment cycles. Similarly, repeated exposure to 1 Gy radiation alone did not impair the self-renewal capacity of BE(2)-C cells (Fig. 6M).

In summary, these data highlight the markedly increased expression of DNA-PKcs in NB cells following chronic irradiation, demonstrating that NB cells rely on DNA-PKcs to deal with sustained radiation-induced stress (Fig. 6N). This dependency likely underpins the complete loss of self-renewal observed in the IR/peposertib combination group after five treatment cycles. Together, these findings highlight the essential role of DNA-PKcs in modulating NB therapeutic response and relapse capacity. DNA-PKcs inhibition combined with targeted DNA-damaging strategies, enhances therapeutic efficacy, promotes tumor regression, and disrupts the self-renewal capacity of metastatic cancer cells.

## 3. Discussion

Neuroblastoma is a developmental tumor of early childhood with significant clinical heterogeneity, ranging from spontaneous regression to rapid progression and patient death [52,53]. While substantial progress has been achieved for low-risk patients, those with HR-NB have poor outcomes despite aggressive multimodal treatments. Substantial efforts have been made to develop targeted therapies for HR-NB patients; however, progressive and relapsed disease particularly within metastatic niches remains a key driver of mortality. In contrast to low somatic mutational burden, most tumors from HR-NB patients are characterized by abundant structural chromosomal abnormalities. In addition to *MYCN* amplification, several other genome alterations, including chromosome 1p and 11q deletion or 17q gain, are associated with HR-NB. Since NB is a copy number driven tumor (C class), identifying targeted inhibitors that exploit its unique features is quite challenging [54–57]. Although ALK activation, either by chromosomal translocations or by point mutations, has been identified in 9% of all NB (strongly correlated with *MYCN* amplification), the improved initial response to multikinase inhibitors has been demonstrated to diminish, likely due to tumor cell adaption through alternative signaling pathways [57–59]. While the precise mechanisms underlying these alterations are still unclear, genomic profiling studies suggest that these complex patterns can be correlated with defects in maintenance genomic integrity. Chromothripsis, the extensive focal genomic shattering and rejoining observed in 18% of HR-NBs, has been shown to be caused by deficient DNA repair systems [60,61]. Moreover, chromosome 11q deletion has been reported in up to 48% of NB cases with distinct genetic subtypes. Given that this region contains genes involved in DSBs repair, it highlights the essential role of DNA repair in HR-NB by regulating the DDRs and activating various cell cycle checkpoints and pathways [54,57, 62–64].

DNA repair dysfunction in tumor cells represents a potential therapeutic venue and since it is associated with HR-NB, targeting its components would be a potential therapeutic approach that could help minimize side effects and the development of drug resistance in NB [29–31]. Through a comprehensive analysis of TMAs and NB cohort datasets, we demonstrated that significant upregulation of DNA-PKcs is associated with poor survival and adverse clinical features. This finding is particularly noteworthy given its pronounced upregulation in disseminated tumor cells (DTCs), whose transcriptional and molecular characteristics remain poorly defined despite the substantial therapeutic challenges they present. Moreover, single-cell RNA-seq profiling of patient samples revealed selective *PRKDC* overexpression in mesenchymal-like MES-NB2 metastatic clusters, reinforcing the notion that DNA-PKcs may support the survival and expansion of metastasis-driving subpopulations. Collectively, these results implicate DNA-PKcs as a critical mediator of metastatic niche establishment and persistence during relapse and advanced disease progression in NB. Supporting our finding, recent genomic studies have characterized different NB risk groups based on their genomic alterations providing valuable insights into the mechanisms underlying NB evolution at advanced stages. These studies indicate that DNA damage footprints, resulting from oxidative and replication stress, as well as DNA repair dysfunction, are enriched in HR-NB patients with poor survival outcomes [31,65]. Our study further highlights that elevated *PRKDC* expression contributes to NB progression and impacts response to DNA-damaging therapies, as validated by both *in vitro* and *in vivo* studies. In concordance with our findings, a recent CRISPR screening study conducted at St. Jude Children’s Research Hospital identified *PRKDC* among the top druggable targets that enhance NB sensitivity to common chemotherapeutic drugs [41]. Notably, a recent *in vivo* study using an *MYCN* transgenic zebrafish model demonstrated the critical role of destructive mutations in DNA repair genes in NB tumorigenesis. This compelling functional evidence highlighted the importance of DSB repair pathway genes, such as *BARD1*, in mediating DDR in NB, and their contribution to metastasis and drug sensitivity, underscoring their potential as high-priority targets for HR-NB treatment [66]. These findings suggest that the effective response of NB cells to DNA damaging therapies, substantially influenced by the capacity of these cells to recruit and activate DNA repair pathways, allowing them to repair damaged DNA and survive [31,67]. Given our findings indicating the high sensitivity of NB cells to DNA-PKcs inhibition, our study further emphasizes the significant role of DNA-PKcs in aggressive NB and underscoring the importance of pursuing targeted therapies.

Radiation therapy is a fundamental practice in the treatment of HR-NB leveraged for both post-operative local control and treatment of relapsed/refractory metastatic disease [1,6]. The metastatic burden of HR-NB is a significant clinical challenge and most commonly occurs to the bone marrow, bone, liver, and lymph nodes in order of decreasing frequency. In this study, we utilized a liver metastasis model to assess whether DNA-damaging therapies, particularly localized radiotherapy, could adversely affect normal tissue surrounding metastatic tumors, potentially restricting their clinical applicability. NB liver metastases also pose a significant clinical concern being a common site of metastasis present in approximately 10% of children with NB [68]. Combination radiotherapy effectively curtailed the progression of aggressive NB metastases and achieved complete eradication of metastatic tumors in regions exposed to maximal radiation doses. The absence of adverse effects in normal tissue supports the safe expansion of combination radiotherapy to other metastatic NB sites, increasing its clinical relevance. Pairing low-dose radiotherapy or stereotactic body radiation therapy with peposertib can further broaden the clinical impact of DNA-PKcs inhibitors. Given that a significant proportion of children with NB experience relapsed or refractory disease in bone and bone marrow, our research group is actively working to develop reliable models of NB bone and bone marrow metastases. This effort aims to determine whether the DNA-PKcs radio sensitization principles identified in this study can be broadly applied to other metastatic niches, potentially expanding therapeutic strategies for HR-NB.

Combining peposertib with doxorubicin yielded potent anti-cancer effects *in vitro* and *in vivo* that were overshadowed by toxicity concerns, prompting early study discontinuation. Microscopic analysis of the liver and lungs revealed minimal impact from the combination therapy, with the primary concern centered on excessive gastrointestinal toxicity. As a member of the phosphatidylinositol 3-kinase-related kinase (PIKK) family, DNA-PKcs shares characteristics with PI3K inhibitors, whose clinical applications have been largely limited due to associated gastrointestinal toxicities [69]. While peposertib alone does not induce detectable gastrointestinal toxicity, adverse effects emerged when combined with Top II inhibitors. To effectively translate the benefits of DNA-PKcs inhibition into clinical practice, it is essential to minimize gastrointestinal toxicity by limiting systemic chemotherapy exposure and instead employing targeted DNA-damaging strategies, such as radiation therapy or proton therapy. Another key approach to reducing toxicity involves optimizing drug administration timing, ensuring DNA-PKcs inhibition occurs only when biologically necessary to prevent adverse effects. Our findings indicate that DNA-PKcs inhibition must be initiated within 4 h post-radiotherapy; otherwise, the therapeutic efficacy of the combination treatment is significantly compromised. The optimal approach involves delivering the DNA-PKcs inhibitor to tumor tissue just before radiation therapy and ensuring its sustained presence for up to 24 h to maximize therapeutic efficacy. A brief but precisely timed inhibition of DNA-PKcs following radiotherapy is sufficient to achieve a strong therapeutic effect, reducing toxicity and minimizing patient exposure while maintaining treatment efficacy. Recurrent and refractory bony HR-NB lesions are often treated with external beam radiation to optimize pain control, reduce imminent life-threating risk and optimize quality of remaining life. Optimizing radiation response with a novel agent without significant expected toxicity is clinically appealing in the recurrent/refractory HR-NB population given that these patients are heavily pre-treated with multimodal anticancer therapies.

A limitation of this study is the focus on a liver metastasis model for efficacy testing. Although the liver is a common site of spread—affecting roughly 20–30% of patients with metastatic neuroblastoma—the majority of metastases occur in bone and bone marrow. The synergy observed between radiotherapy and peposertib is likely to be preserved in those sites, although confirmation in bone and bone-marrow metastasis models is required. In addition, the radiation regimens used here differ from clinical practice; while the pronounced tumor response at 1–2 Gy raises the possibility of clinically relevant dose-reduction strategies, confirmation will require experiments that replicate current clinical protocol.

In conclusion, the poor overall therapeutic success and long-term side effects of intensive chemotherapy in HR-NB patients underscore the necessity of introducing more targeted treatments that exploit the molecular characteristics of NB tumors to overcome therapeutic resistance. Our findings demonstrate the central role of DNA-PKcs in HR-NB, revealing a critical dependence of NB cells on this gene and positioning it as a compelling target for precision therapy. The enhanced efficacy of Top II inhibitors and radiotherapy in the presence of DNA-PKcs inhibition underscores the essential function of this protein in NB response, reinforcing the potential of selective DNA-PKcs inhibition as an effective strategy for maximizing therapeutic impact in HR-NB—especially when synergistically combined with radiation therapy to drive metastatic tumor regression.

## 4. Materials and methods

### Cell culture and drug preparation.

*MYCN*-amplified human NB cell lines (BE(2)-C (CVCL_V006), IMR-32 (CVCL_0346), LAN-1 (CVCL_1827) and SK-N-DZ (CVCL_1701)) and *MYCN*-non-amplified cells (SK-N-AS (CVCL_1700), and SK-N-SH (CVCL_0531)) were purchased from ATCC. IMR-32 and LAN-1 cells were cultured in Eagle’s Minimal Essential Medium (EMEM) because when isolated, the IMR-32 cells were mixed with large hyaline fibroblasts which would predominate in the culture mixture if a media other than EMEM were used. BE(2)-C, SK-NA-S and SK-N-SH were cultured in RPMI media. SK-N-DZ cells were grown in a 1:1:1 mixture of EMEM and Dulbecco’s Modified Eagle’s Medium and Ham’s F-12 Nutrient Mixture (DMEM/F12) Medium. All media employed in this study were supplemented with 10% fetal bovine serum (FBS), along with 100 units of penicillin and 100 μg of streptomycin/ml. The cells were sustained in a humidified incubator at 37 °C with 5% CO_2_. The stock solutions of etoposide (#12092), doxorubicin hydrochloride (#15007) and peposertib were prepared in DMSO (Cayman Chemical, MI). Peposertib was received through The Cancer Therapy Evaluation Program. The final DMSO concentration did not exceed 0.1% (v/v) to minimize cytotoxic effects, ensuring that it did not change the cell responses.

### Viral transductions and stable knockdown (shRNA transfection).

Lentiviral plasmids encoding shRNA *PRKDC* (SC-35200) and non-target control (NTC) shRNA were obtained from Santa Cruz Biotechnology (Santa Cruz, CA). BE(2)-C cells were seeded in a 12-well plate 24 h prior to viral infection. At 70% confluency, the media were removed, and 1 ml of a mixture of complete medium containing Polybrene (sc-134220) at a final concentration of 5 μg/ml was added to each well. Then, 5 μl of lentiviral shRNA lentiviral particles were added to each well. Stable clones expressing the shRNA were selected by culturing in medium containing 2 μg/ml puromycin dihydrochloride.

### Cell proliferation and clonogenic assays.

Cell proliferation assays were performed as previously outlined [70] using the Sulforhodamine B (SRB; G-Biosciences, Overland, MO, #786–21) assay. Briefly, 5000 cells were seeded in 96-well plates in the presence of varying concentrations of etoposide or doxorubicin alone or in combination with peposertib (1 μM). After 4 and 6 d, BE(2)-C and SK-N-DZ cells were fixed with 10% trichloroacetic acid, respectively. The cells were stained with 0.4% SRB, washed with 1% acetic acid, and solubilized with 10 mM Trizma base solution (pH 10.5–12.0). The optical density of the protein-bound dye dissolved in the solubilization buffer solution was measured at 565 nm. Experiments were conducted in six replicates and repeated at least twice. IC_50_ was determined using PRISM GraphPad. IC_50_was defined as the concentration of the compound at which tumor cell growth was inhibited by 50% compared to the control.

Colony formation assay was performed to evaluate whether stable *PRKDC* gene inhibition impacts the survival rate of cancer cells seeded at a very low density (1000 cells/well). Similarly, the inhibitory effects of Top II inhibitors in combination with peposertib were evaluated. BE(2)-C and SK-N-DZ cells were plated into each well of a six-well plate and treated with DMSO, peposertib (1 μM), etoposide (BE(2)-C = 0.5 μM and SK-N-DZ = 0.1 μM), and a combination of the two. Similarly, BE(2)-C and SK-N-DZ cells were treated with 75 and 10 nm of doxorubicin, respectively, both alone and in combination. Media were changed after 48 h, and cells were maintained in standard medium. After 14 d, colonies were fixed and stained with SRB buffer using the method described above. Subsequently, the dye was resolubilized with 2 ml of dye release solution. Dye solution was diluted 1:3 using Trizma base solution and added to the wells of a 96-well plate, then absorbance was measured using a plate reader at a wavelength of 565 nm. Accordingly, the same treatments were applied to evaluate the inhibitory effects of etoposide and doxorubicin on the growth of NTC and *PRKDC* stable shRNA BE(2)-C knockout cells.

### Cell apoptosis assay using cell death ELISA.

The cytoplasmic histone-associated DNA fragments were measured using a cell death ELISA kit (Roche Applied Science, Penzberg, Germany). BE(2)-C and SK-N-DZ cells were treated with different concentrations of etoposide (0.5 μM and 0.1 μM, respectively) and doxorubicin (250 nM and 25 nM, respectively), both alone and in combination with peposertib (1 μM). After 48 h, DNA fragmentation was quantified and plotted according to the kit manufacturer’s instructions.

### Western blot.

To assess protein expression changes, Western blot (WB) analysis was conducted using target protein’s specific antibodies. NB cells (10^6^ cells/well) were seeded into a 6-well plate and subjected to treatment corresponding to each experimental setting. At the appropriate time points, cells were collected, and protein extraction was performed using RIPA lysis buffer. The protein concentration was quantified using a Bradford assay, and 80 μg of protein lysate was subsequently subjected to standard WB gel electrophoresis. The blots were exposed to pDNA-PKcs (AB_2771465), DNA-PKcs (AB_3714735), N-Myc (AB_2799400), c-PARP (AB_304666), and CyclinD1 (AB_2750906) antibodies overnight and protein bands were visualized. β-actin (AB_476744) was used as loading control and the relative expression levels of the proteins were quantified using ImageJ (SCR_003070).

### Tissue Microarrays and immunohistochemistry (IHC).

Tissue microarrays (TMAs) comprising neuroblastoma and peripheral nerve tissues were utilized to evaluate *PRKDC* gene expression in patient samples. The array (NB642c) consisted of 27 NB cases and 5 peripheral nerve tissue used as normal controls, with each case represented by duplicate cores measuring 1.5 mm in diameter. Immunostaining was carried out using standardized protocols, as previously described [71]. A pathologist assessed the IHC slides, scoring both the extent and intensity of staining using the semi-quantitative seven-point system [72]. This scoring method evaluates the proportion of positive cells (none = 0; <10% = 1; 10–50% = 2; >50% = 3) and staining intensity (none = 0; weak = 1; intermediate = 2; strong = 3), with the final score obtained by summing these two values.

### DNA damage assay by immunofluorescence.

Confocal imaging of phosphorylated γ-H2AX (Ser139) specific Ab (AB_2783871) was performed to measure induced DSBs in NB cells. BE(2)-C and SK-N-DZ cells were grown (200 × 10^3^) on coverslips overnight and incubated with DMSO, 2 μM, and 0.5 μM etoposide, alone and in combination with 1 μM peposertib, respectively. Both cells were treated with doxorubicin 250 μM, using the same procedure, both alone and in combination. Following 24 h after each treatment, cells were washed with FBS and then fixed in 4% paraformaldehyde. Cells were permeabilized with 0.5% Triton X-100 and blocked with 5% normal horse serum 2.5%. Then, cells were incubated with *anti*-*γ*-H2AX (Ser139) Ab overnight at 4 °C, washed with PBS and incubated with VectaFluor Anti Mouse secondary antibodies (red, AB_2336783) for 30 min at 37 °C in the dark. DAPI staining was used to stain cell nuclei (blue), and the cells were imaged using immunofluorescence microscopy.

### Radiotherapy in vitro.

Cells were irradiated at the X-ray Service Center in the University of Kentucky, Department of Toxicology and Cancer Biology. Briefly, cells were placed on the X-RAD 225XL orthovoltage X-ray platform (Precision X-ray, Madison, CT) with an aluminum filter and received a dose rate of approximately 2.0 Gy/min. Absorbed doses were calculated with consideration of the impact of backscattering as previously described [73].

### Doxorubicin and peposertib administration in vivo.

Fresh solution of doxorubicin hydrochloride in 0.9% sodium chloride was prepared for each injection. Mice received 200 μl intravenous injection of doxorubicin hydrocholoride after peposertib administration by oral gavage. Powdered peposertib was diluted in 0.5% Methocel, 0.25% Tween 20, 300 mM Na-Citrate pH 2.5 before every therapy. Briefly, citric acid monohydrate (6.3 g) was diluted in DDI water (50 ml) and adjusted to pH 2.5 with NaOH cookies (~4). Next, Methocel (0.5 g) was added slowly into stirred solution to avoid clumping. The solution volume was adjusted to 100 ml and left mixing at 80 °C overnight. Next day, the solution was cleared with Tween 20 (250 μl), aliquoted and stored at 4 °C for further use. For IR, peposertib was administered by oral gavage 30 min before each radiotherapy session. In the IR alone group, local 2 Gy radiation was administered daily for 4 d. In the combined peposertib + IR group, peposertib was administered at 100 mg/kg by oral gavage once daily for 4 d, and irradiation was performed 30 min after peposertib drug administration (2 Gy × 4 d).

### Study approval.

Animal experiments were approved by the Institutional Animal Studies Committee at University of Kentucky. Animals received humane care in compliance with the Guide for the Care and Use of Laboratory Animals (National Academies Press, 2011) and with the Principles of Laboratory Animal Care formulated by the National Society for Medical Research. Human protocols were approved by the Institutional Review Board at University of Kentucky (no. 2021–3847).

### Animal Demographics.

Both male and female animals were included in the study, and similar findings are reported for both sexes. All animals were 8 weeks old and weighed approximately 25 g at the beginning of the experiment.

### Animal experiments.

All animal experiments were performed at the University of Kentucky. NOD.Cg-*Rag1*^*tm1Mom*^ *Il2rg*^*tm1Wjl*^/SzJ strain mice (IMSR_JAX:007799) were used to establish the BE(2)-C liver metastasis model. First, BE(2)-C cells were transfected with pEGFP-N1 plasmid (Clontech Laboratories, Mountain View, CA; #6085–1, Addgene_172284) and firefly luciferase (GenTarget, San Diego, CA; #LVP1222-PBS). Next, mice were anesthetized using isoflurane (3% in oxygen at 0.6 L/min flow rate), placed on a prewarmed (42 °C) surgical bed and injected intravenously with cells (100 μl, 1 × 10^6^ cells, PBS).

Twenty NOD.Cg-Rag1tm1Mom Il2rgtm1Wjl/SzJ mice (n = 5 per group) were randomized to receive control (gavage; 200 μl 0.5% Methocel, 0.25% Tween 20, 300 mM Na-Citrate pH 2.5; no radiotherapy), peposertib (gavage, 100 mg/kg in 200 μl of 0.5% Methocel, 0.25% Tween 20, 300 mM Na-Citrate pH 2.5), every 24 h, 4 treatments), radiotherapy (2 Gy, every 24 h, 4 treatments), or combination modality (gavage; 100 mg/kg in 200 μl of 0.5% Methocel, 0.25% Tween 20, 300 mM Na-Citrate pH 2.5) administered 30 min prior to radiotherapy, every 24 h for a total of 4 treatments). Mice were randomized 6 d after cancer cell injection and treated as defined by treatment groups (n = 5). Two weeks after cancer cell injection, control and peposertib groups were euthanized due to metastatic burden. IR and IR + peposertib groups were imaged 23 d after cancer cell injection to quantify metastatic burden by bioluminescent optical imaging. The total bioluminescent emission intensity reflected remaining active disease in the tissues.

### Radiotherapy in vivo.

Prior to the radiation procedures, mice were anesthetized by ip administration of (10 mg/kg; AnaSed xylazine, NADA 139–236, Akorn, IL) and ketamine (100 mg/kg; Ketathesia Ketamine hydrochloride, Henry Schein Animal Health, Dublin, OH). The metastatic tumor sites of mice were precisely positioned on the X-RAD 225 XL platform for the X-ray exposure with the adjustable collimator [74,75]. The tumor sites were then irradiated with a copper filter and a dose rate of approximately 1.6 Gy/min.

### Bioluminescent imaging in vivo.

The total bioluminescent emission intensity reflected remaining active disease in the tissues. Bioluminescent and GFP fluorescent imaging was carried out using a Lago SII station (Spectral Instruments Imaging; Tucson, AZ). Composite images obtained were comprised of black and white digital photos with an overlay of images reflecting fluorescent activity. Bioluminescence was quantified by drawing a square region of interest that encompassed the liver area in the abdomen and measuring the signal within that ROI. The density map, measured as photons/s/cm^2^/steradian, was created using the Aura software (Spectral Instruments Imaging; Tucson, AZ) and represented as a color gradient centered at the maximal spot. Panoramic GFP fluorescence imaging was performed using an LT-9500 Illumatool/TLS (Lightools Research, Encinitas, CA), equipped with an excitation source (470 nm) and filter plate (515 nm).

### Establishment and characterization of doxorubicin and radiation-resistant cell lines.

Doxorubicin- and radiation-resistant BE(2)-C cell lines were established through repeated exposure to doxorubicin, IR, or their combination with peposertib. BE(2)-C cells were seeded at a density of 1000 cells per well and treated with 50 nM doxorubicin, either alone or in combination with 1000 nM peposertib. In parallel, cells were pretreated with either DMSO or 1000 nM peposertib for 30 min prior to exposure to 1 Gy IR. After 48 h, the medium was replaced with fresh growth medium, and the cells were incubated for an additional 12 d to allow colony formation. This cycle was repeated 5 times, ensuring consistent cell density and treatment conditions throughout. Cells that survived 5 cycles of 1 Gy IR were designated as chronic IR survivor cells. In contrast, acute IR survivor cells were derived from parental colonies exposed once to 5 Gy IR and cultured under standard conditions until confluence was reached.

### Single-nucleus RNA-Sequencing (snRNA-seq) and analysis.

Human NBs located at primary adrenal region (n = 7) and lymph node metastases (n = 5) post-induction therapy were procured with consent from patients with intermediate to high-risk NBs under approval and oversight by the Institutional Review Board committees of Cincinnati Children’s Hospital Medical Center. Briefly, snap-frozen human NB specimens were lysed in ice-cold nuclei lysis buffer (Nuclei Extraction Buffer (Miltenyi Biotec, Baltimore, MD) containing DTT (Sigma-Aldrich, St. Louis, MO) and RNAse inhibitor (Sigma-Aldrich)) for 5 min on a gentle MACS Octo Dissociator at 4 °C with agitation. After lysis, samples were filtered through a 70-μm cell strainer, washed with lysis buffer and centrifuged for 5 min at 500×*g* at 4 °C. Nuclei pellet was resuspended with ice-cold 0.1% BSA in PBS with RNAse inhibitor, filtered through a 30-μm cell strainer and centrifuged at 500×*g* at 4 °C for 5 min. Next, nuclei were permeabilized by resuspending the pellet in 0.1x lysis buffer (10 mM NaCl, 10 mM Tris 7.4, 3 mM MgCl2, 0.1% IGEPAL CA-63, 0.01% Digitonin, 1% BSA, 1 mM DTT, 1 U/μL of Protector RNase inhibitor (Sigma)) and incubated on ice for 2 min. Then, 1 mL Wash Buffer (10 mM NaCl, 10 mM Tris 7.4, 3 mM MgCl2, 0.1% Tween-20, 1% BSA, 1 mM DTT, 1 U/μL of Protector RNase inhibitor (Sigma)) was added. The nuclei were centrifuged at 500×*g* for 5 min and the supernatant was discarded. The nuclei pellet was resuspended in diluted nuclei buffer (1x Nuclei buffer Multiome kit (10x Genomics, Oxford, UK)), 1 mM DTT, 1 U/μL of Protector RNase inhibitor (Sigma) according to the 10X Genomics protocol for Nuclei Isolation from Complex Tissues for Single Cell Multiome ATAC + Gene Expression Sequencing (RRID:SCR_015980).

Single-nuclei libraries were generated using the 10x Chromium Single-Cell Instrument and NextGEM Single Cell Multiome ATAC + Gene Expression kit (10x Genomics) according to the manufacturer’s protocol. All 10x Multiome GEX libraries were sequenced on NovaSeq6000 instruments to a target depth of 250 million read pairs per sample.

For computational analysis, demultiplexed scRNA-seq FASTQ files were input into the Cell Ranger ARC pipeline (version 2.0.0) from 10x Genomics to align reads to the hg38 human reference sequence and generate barcoded count matrices of gene expression and ATAC data. scRNA-seq and snMultiome-seq data was analyzed with the open-source Seurat (SCR_016341) [76] and ArchR (SCR_020982) packages [77] implemented in the R computing environment. For each sample dataset, unsupervised clustering was performed using R package Seurat (version 4) (https://www.cell.com/cell/fulltext/S0092-8674(21)00583-3?_returnURL=https%3A%2F%2Flinkinghub.elsevier.com%2Fretrieve%2Fpii%2FS0092867421005833%3Fshowall%3Dtrue). Based on the distribution of cells ordered by percentage of mitochondrial genes and detected gene numbers, we excluded cells with either more than 9000 detected genes or less than 200, and mitochondrial content of more than 20%. Following normalization using Seurat’s NormalizeData function, highly variable genes were identified and used for principal components analysis. Then, we performed clustering using graph-based clustering and visualized using Uniform Manifold Approximation and Projection (UMAP) with Seurat function RunUMAP. To identity the cell type in tumor sample datasets, we input marker gene lists generated by Seurat FindMarkers function in Toppgene (SCR_005726) to identify the top cell type makers or cell identities. Differentially expressed genes (DEGs) in a given cell type compared with all other cell types were determined with the FindAllMarkers function in the Seurat package.

## Supplementary Material

Supplmental Material

## Data Availability

We applied R2 Genomics Analysis and Visualization Platform (https://hgserver1.amc.nl/cgi-bin/r2/main.cgi), which contained comprehensive clinical and prognostic information, to analyze and visualize gene expression data from different NB cohorts (Ambros, Kocak, and SEQC). Gene expression data from Kocak, and SEQC were obtained and survival analysis was performed within the R2 Genomics platform. Kaplan-Meier survival curves were generated to compare overall survival between high and low *PRKDC* expression groups, stratified by the median gene expression level. The raw log-rank test used to assess statistical significance. A p-value less than 0.05 was considered statistically significant. Data presented in this study were analyzed using GraphPad Prism (version 9.5.1, SCR_002798). Statistical comparisons between two groups were performed using two-tailed unpaired Student’s t-tests, assuming normal distribution and equal variances. For comparisons involving more than two groups, one-way ANOVA was used, followed by Tukey’s test. A significance level was set at: *p < 0.05, **p < 0.01, ***p < 0.001, ****p < 0.0001. All experiments were conducted in triplicate, and results are presented as mean ± SD.

## Appendix A. Supplementary data

Supplementary data to this article can be found online at https://doi.org/10.1016/j.canlet.2026.218383.

## Term: Definition

Neuroblastoma (NB): A pediatric cancer that develops from immature nerve cells, commonly found in and around the adrenal glands
MYCN amplification: A genetic alteration where multiple copies of the MYCN gene are present, associated with aggressive forms of neuroblastoma
shRNA (short hairpin RNA): A synthetic RNA molecule used to silence gene expression via RNA interference
PRKDC: A gene encoding DNA-dependent protein kinase catalytic subunit (DNA-PKcs), crucial for DNA damage repair
SRB assay: A colorimetric assay used to measure cell density and proliferation by staining cellular proteins
IC50: The concentration of a drug that inhibits a biological process by 50%
Cell death ELISA: A quantitative assay detecting DNA fragmentation, a hallmark of apoptosis
Topoisomerase II (Top II) inhibitors: Drugs such as etoposide and doxorubicin that disrupt DNA replication and transcription by inhibiting topoisomerase II
γ-H2AX: A phosphorylated form of histone H2AX, used as a marker for DNA double-strand breaks
IR (Ionizing Radiation): High-energy radiation used in cancer therapy to damage DNA and kill cancer cells
