# Supplementary material for: DNA-PKcs promotes therapy resistance and metastatic recurrence in neuroblastoma
Source: Cancer Lett. Author manuscript; Available in PMC 2026 Apr 20. (PMC13093346; doi:10.1016/j.canlet.2026.218383)
Supplement: Supplmental Material [file NIHMS2159352-supplement-Supplmental_Material.pptx]

## Slide 1
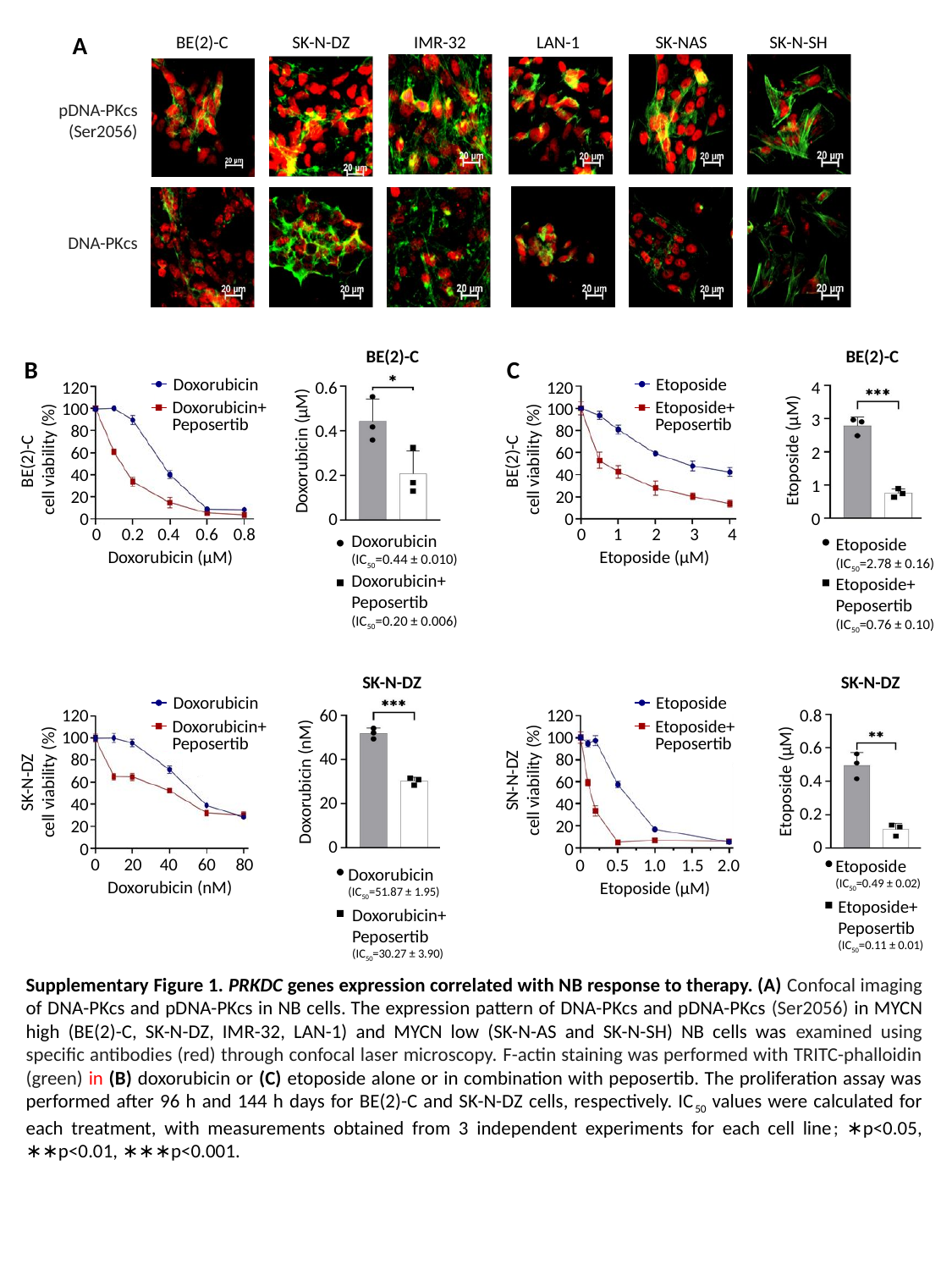

A
BE(2)-C
SK-N-DZ
IMR-32
LAN-1
SK-NAS
SK-N-SH
pDNA-PKcs (Ser2056)
DNA-PKcs
BE(2)-C
BE(2)-C
B
C
Doxorubicin
Doxorubicin+
Peposertib
Etoposide
Etoposide+
Peposertib
120
0.6
120
4
100
100
3
80
0.4
80
BE(2)-C
cell viability (%)
BE(2)-C
cell viability (%)
Doxorubicin (μM)
Etoposide (μM)
60
60
2
40
0.2
40
1
20
20
0
0
0
0
0
0.2
0.4
0.6
0.8
0
1
2
3
4
Doxorubicin
(IC50=0.44 ± 0.010)
Etoposide
(IC50=2.78 ± 0.16)
Doxorubicin (μM)
Etoposide (μM)
Doxorubicin+
Peposertib
(IC50=0.20 ± 0.006)
Etoposide+
Peposertib
(IC50=0.76 ± 0.10)
SK-N-DZ
SK-N-DZ
Doxorubicin
Doxorubicin+
Peposertib
Etoposide
Etoposide+
Peposertib
120
60
120
0.8
100
100
0.6
40
80
80
SN-N-DZ
cell viability (%)
SK-N-DZ
cell viability (%)
0.4
60
60
Doxorubicin (nM)
Etoposide (μM)
20
40
40
0.2
20
20
0
0
0
0
0
20
40
60
80
0
0.5
1.0
1.5
2.0
Etoposide
(IC50=0.49 ± 0.02)
Doxorubicin
(IC50=51.87 ± 1.95)
Doxorubicin (nM)
Etoposide (μM)
Etoposide+
Peposertib
(IC50=0.11 ± 0.01)
Doxorubicin+
Peposertib
(IC50=30.27 ± 3.90)
Supplementary Figure 1. PRKDC genes expression correlated with NB response to therapy. (A) Confocal imaging of DNA-PKcs and pDNA-PKcs in NB cells. The expression pattern of DNA-PKcs and pDNA-PKcs (Ser2056) in MYCN high (BE(2)-C, SK-N-DZ, IMR-32, LAN-1) and MYCN low (SK-N-AS and SK-N-SH) NB cells was examined using specific antibodies (red) through confocal laser microscopy. F-actin staining was performed with TRITC-phalloidin (green) in (B) doxorubicin or (C) etoposide alone or in combination with peposertib. The proliferation assay was performed after 96 h and 144 h days for BE(2)-C and SK-N-DZ cells, respectively. IC50 values were calculated for each treatment, with measurements obtained from 3 independent experiments for each cell line; ∗p<0.05, ∗∗p<0.01, ∗∗∗p<0.001.

## Slide 2
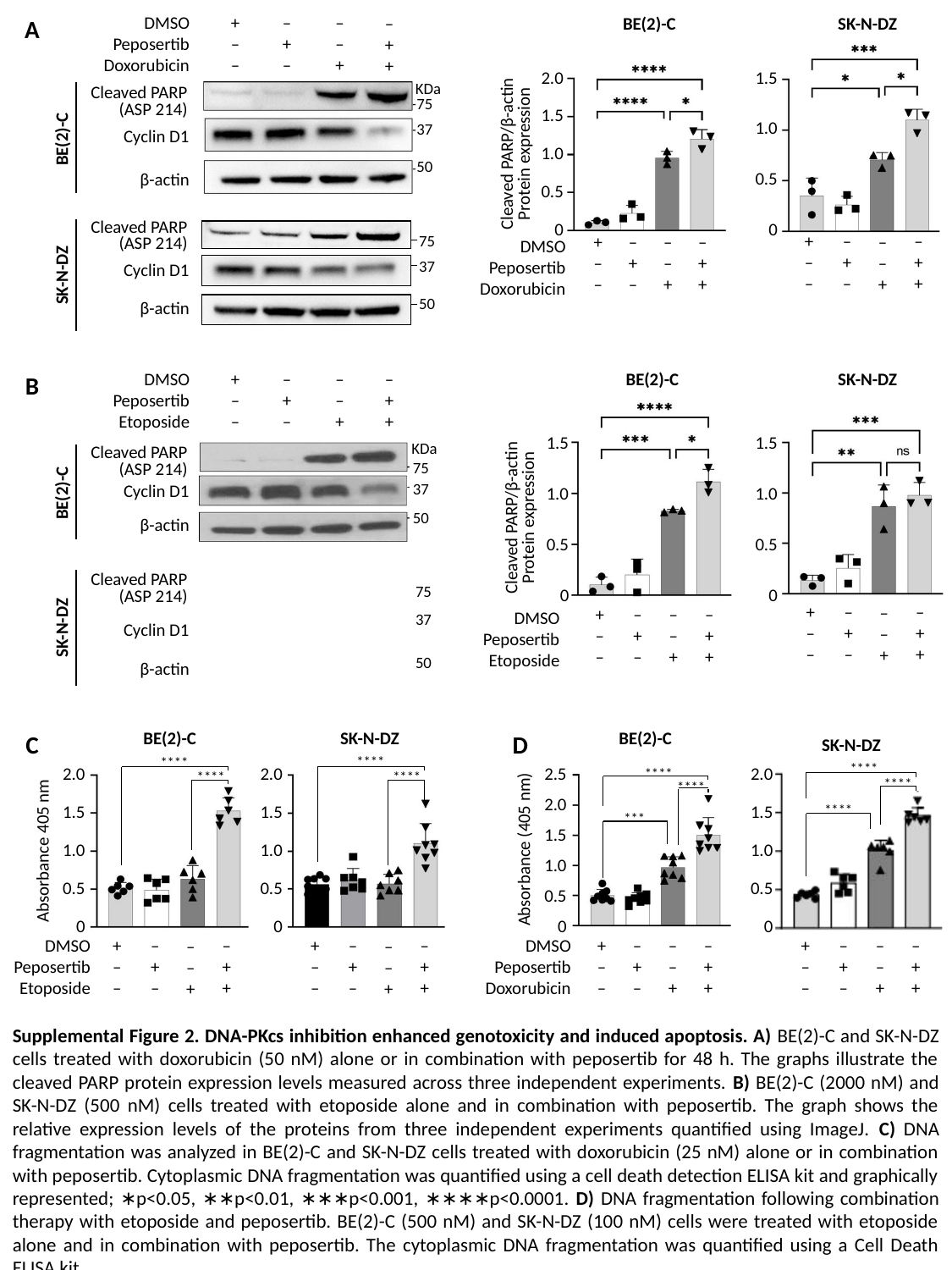

DMSO
Peposertib
Doxorubicin
+
–
–
–
+
–
–
–
+
–
+
+
BE(2)-C
SK-N-DZ
A
2.0
1.5
KDa
Cleaved PARP
(ASP 214)
75
1.5
1.0
37
BE(2)-C
Cyclin D1
Cleaved PARP/β-actin Protein expression
1.0
50
0.5
β-actin
0.5
0
0
Cleaved PARP
(ASP 214)
+
–
–
–
+
–
–
+
+
+
–
–
–
+
–
–
+
+
–
–
+
–
–
+
75
DMSO
Peposertib
Doxorubicin
37
Cyclin D1
SK-N-DZ
50
β-actin
DMSO
Peposertib
Etoposide
+
–
–
–
+
–
–
–
+
–
+
+
BE(2)-C
SK-N-DZ
B
1.5
1.5
KDa
Cleaved PARP
(ASP 214)
75
BE(2)-C
1.0
1.0
37
Cyclin D1
Cleaved PARP/β-actin Protein expression
50
β-actin
0.5
0.5
Cleaved PARP
(ASP 214)
0
0
75
+
–
–
–
+
–
–
+
+
–
–
+
+
–
–
–
+
–
–
+
+
–
–
+
DMSO
Peposertib
Etoposide
37
SK-N-DZ
Cyclin D1
50
β-actin
BE(2)-C
SK-N-DZ
BE(2)-C
C
D
SK-N-DZ
****
****
****
****
2.0
2.0
2.5
2.0
Absorbance (405 nm)
****
****
***
****
****
2.0
1.5
1.5
1.5
****
1.5
1.0
1.0
1.0
Absorbance 405 nm
1.0
0.5
0.5
0.5
0.5
0
0
0
0
DMSO
Peposertib
Etoposide
+
–
–
–
+
–
–
+
+
–
–
+
+
–
–
–
+
–
–
+
+
–
–
+
DMSO
Peposertib
Doxorubicin
+
–
–
–
+
–
–
–
+
–
+
+
+
–
–
–
+
–
–
–
+
–
+
+
Supplemental Figure 2. DNA-PKcs inhibition enhanced genotoxicity and induced apoptosis. A) BE(2)-C and SK-N-DZ cells treated with doxorubicin (50 nM) alone or in combination with peposertib for 48 h. The graphs illustrate the cleaved PARP protein expression levels measured across three independent experiments. B) BE(2)-C (2000 nM) and SK-N-DZ (500 nM) cells treated with etoposide alone and in combination with peposertib. The graph shows the relative expression levels of the proteins from three independent experiments quantified using ImageJ. C) DNA fragmentation was analyzed in BE(2)-C and SK-N-DZ cells treated with doxorubicin (25 nM) alone or in combination with peposertib. Cytoplasmic DNA fragmentation was quantified using a cell death detection ELISA kit and graphically represented; ∗p<0.05, ∗∗p<0.01, ∗∗∗p<0.001, ∗∗∗∗p<0.0001. D) DNA fragmentation following combination therapy with etoposide and peposertib. BE(2)-C (500 nM) and SK-N-DZ (100 nM) cells were treated with etoposide alone and in combination with peposertib. The cytoplasmic DNA fragmentation was quantified using a Cell Death ELISA kit.

## Slide 3
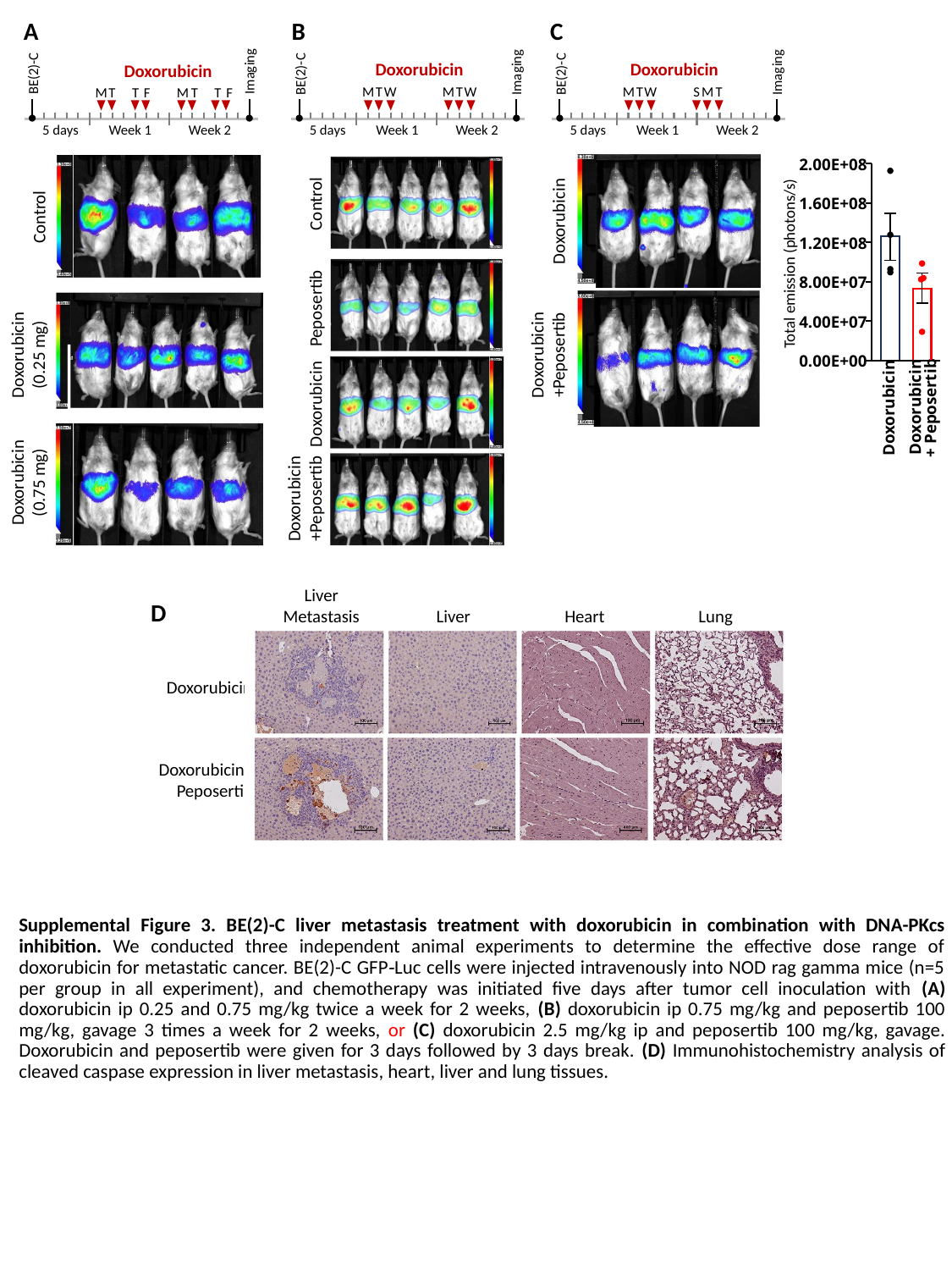

A
B
C
Doxorubicin
Imaging
BE(2)-C
M
T
T
F
M
T
T
F
5 days
Week 1
Week 2
Doxorubicin
Imaging
BE(2)-C
M
T
W
M
T
W
5 days
Week 1
Week 2
Doxorubicin
Imaging
BE(2)-C
M
T
W
S
M
T
5 days
Week 1
Week 2
2.00E+08
Control
Peposertib
Doxorubicin
Doxorubicin
+Peposertib
Doxorubicin
Doxorubicin
+Peposertib
Control
Doxorubicin
(0.25 mg)
Doxorubicin
(0.75 mg)
1.60E+08
1.20E+08
Total emission (photons/s)
8.00E+07
4.00E+07
0.00E+00
Doxorubicin
+ Peposertib
Doxorubicin
Liver Metastasis
Liver
Heart
Lung
Doxorubicin
Doxorubicin+
Peposertib
D
Supplemental Figure 3. BE(2)-C liver metastasis treatment with doxorubicin in combination with DNA-PKcs inhibition. We conducted three independent animal experiments to determine the effective dose range of doxorubicin for metastatic cancer. BE(2)-C GFP‑Luc cells were injected intravenously into NOD rag gamma mice (n=5 per group in all experiment), and chemotherapy was initiated five days after tumor cell inoculation with (A) doxorubicin ip 0.25 and 0.75 mg/kg twice a week for 2 weeks, (B) doxorubicin ip 0.75 mg/kg and peposertib 100 mg/kg, gavage 3 times a week for 2 weeks, or (C) doxorubicin 2.5 mg/kg ip and peposertib 100 mg/kg, gavage. Doxorubicin and peposertib were given for 3 days followed by 3 days break. (D) Immunohistochemistry analysis of cleaved caspase expression in liver metastasis, heart, liver and lung tissues.

## Slide 4
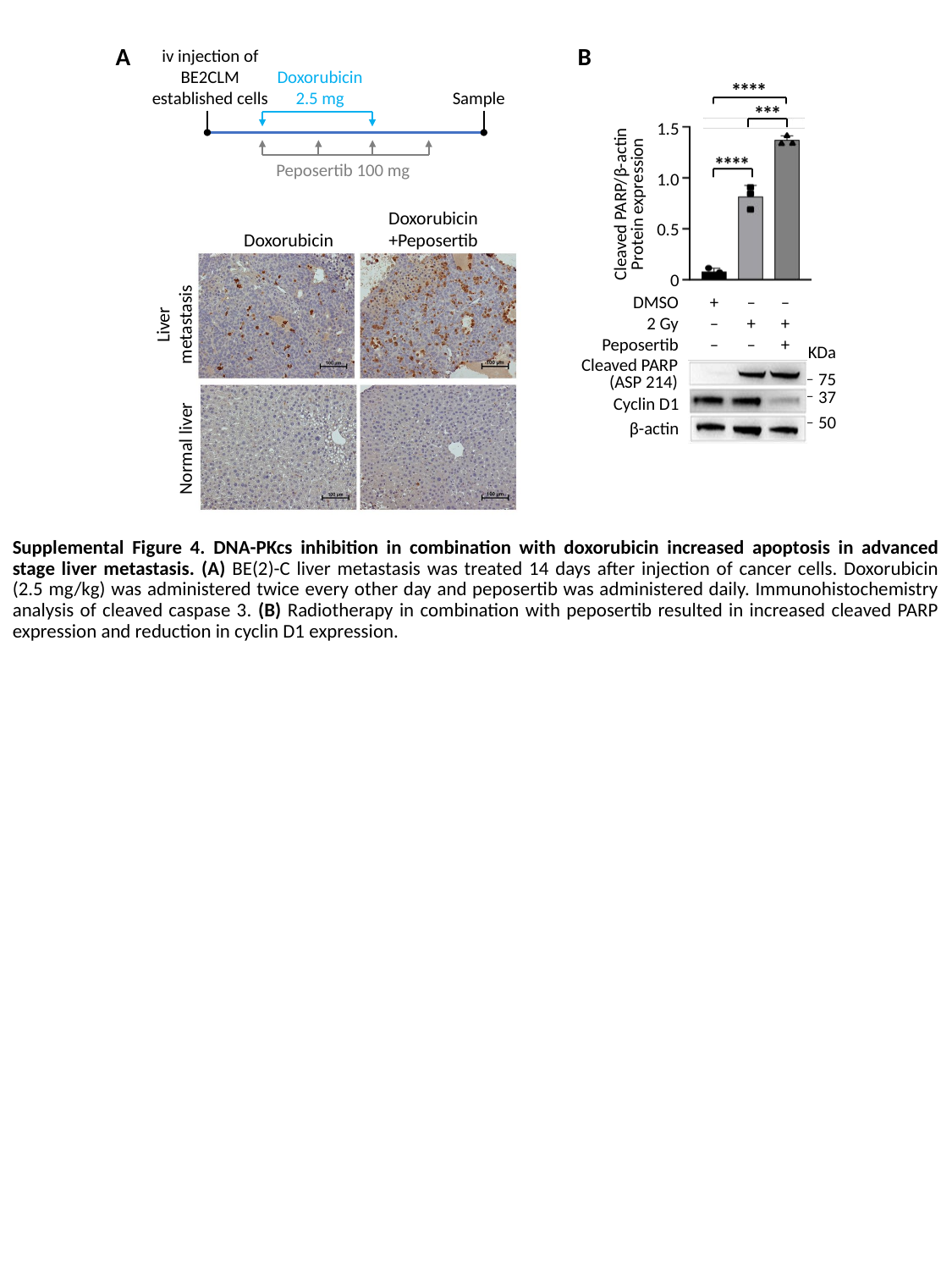

A
B
iv injection of BE2CLM established cells
Doxorubicin
2.5 mg
Sample
Peposertib 100 mg
1.5
1.0
Cleaved PARP/β-actin Protein expression
Doxorubicin +Peposertib
Doxorubicin
Liver metastasis
Normal liver
0.5
0
DMSO
2 Gy
Peposertib
+
–
–
–
+
–
–
+
+
KDa
Cleaved PARP
(ASP 214)
75
37
Cyclin D1
50
β-actin
Supplemental Figure 4. DNA-PKcs inhibition in combination with doxorubicin increased apoptosis in advanced stage liver metastasis. (A) BE(2)-C liver metastasis was treated 14 days after injection of cancer cells. Doxorubicin (2.5 mg/kg) was administered twice every other day and peposertib was administered daily. Immunohistochemistry analysis of cleaved caspase 3. (B) Radiotherapy in combination with peposertib resulted in increased cleaved PARP expression and reduction in cyclin D1 expression.
